# Small Modifications to Network Topology Can Induce Stochastic Bistable Spiking Dynamics in a Balanced Cortical Model

**DOI:** 10.1371/journal.pone.0088254

**Published:** 2014-04-17

**Authors:** Mark D. McDonnell, Lawrence M. Ward

**Affiliations:** 1 Computational and Theoretical Neuroscience Laboratory, Institute for Telecommunications Research, University of South Australia, Mawson Lakes, South Australia, Australia; 2 Department of Psychology and Brain Research Centre, University of British Columbia, Vancouver, British Columbia, Canada; University of Michigan, United States of America

## Abstract

Directed random graph models frequently are used successfully in modeling the population dynamics of networks of cortical neurons connected by chemical synapses. Experimental results consistently reveal that neuronal network topology is complex, however, in the sense that it differs statistically from a random network, and differs for classes of neurons that are physiologically different. This suggests that complex network models whose subnetworks have distinct topological structure may be a useful, and more biologically realistic, alternative to random networks. Here we demonstrate that the balanced excitation and inhibition frequently observed in small cortical regions can transiently disappear in otherwise standard neuronal-scale models of fluctuation-driven dynamics, solely because the random network topology was replaced by a complex clustered one, whilst not changing the in-degree of any neurons. In this network, a small subset of cells whose inhibition comes only from outside their local cluster are the cause of bistable population dynamics, where different clusters of these cells irregularly switch back and forth from a sparsely firing state to a highly active state. Transitions to the highly active state occur when a cluster of these cells spikes sufficiently often to cause strong unbalanced positive feedback to each other. Transitions back to the sparsely firing state rely on occasional large fluctuations in the amount of non-local inhibition received. Neurons in the model are homogeneous in their intrinsic dynamics and in-degrees, but differ in the abundance of various directed feedback motifs in which they participate. Our findings suggest that (i) models and simulations should take into account complex structure that varies for neuron and synapse classes; (ii) differences in the dynamics of neurons with similar intrinsic properties may be caused by their membership in distinctive local networks; (iii) it is important to identify neurons that share physiological properties and location, but differ in their connectivity.

## Introduction

Rapid experimental progress in mapping the functional and structural connections between brain regions (also called the ‘connectome’ [1]–[13]) is delivering critical new understanding about how information processing in the brain is influenced by complex network connectivity [4]. Analysis of connectome data [5]–[7] is increasingly reliant on theoretical insights from the field of network science [8],[9], in which networks are represented by mathematical graph models. The unification of functional neuroanatomy and network science has led to the discovery of significant correlations between abnormal connectivity patterns and several disorders, including epilepsy [10] and schizophrenia [11].

Almost all existing connectome data, however, are at the macroscale of links between brain regions—‘regional connectomes’—whereas fundamental advances in understanding microscale neuronal computation and structure-function relationships will require ‘dense’ (comprehensive) mapping of synaptic connectivity between all individual neurons in a region [2], i.e. ‘neuronal connectomes’ [12]. At present, mapping neuronal connectomes is not feasible experimentally at large enough scales [3],[13] for typical network science approaches to be fully applicable to real data because, in order to avoid finite-size effects, theoretical network science analysis typically requires large scale networks with many thousands of nodes [14]. Despite reports of small and partial neuronal connectomes [15],[16], the only organism whose entire synaptic network has been mapped remains that of the 302 neuron *C. elegans* hermaphrodite worm [6], although a partial connectome of 170 neurons in the *C. elegans* male has now been published [17].

Despite the absence of guiding experimental data, existing network science methods have begun to be applied fruitfully in simulations of hypothesised complex structure within the synaptic connectivity between neocortical or hippocampal neurons [10],[18]–[27]. These simulations produce predictions about the influence of complex network connectivity on spiking patterns, and have begun to cast light on two fundamentally important scientific questions that cannot be answered by current experimental technology:

- What complex structure is embedded in the synaptic networks of small cortical regions?
- How does complex structure in these cortical networks influence neuronal dynamics, both for individual neurons, and collectively in the network?

Fully answering the first question will have to await the maturation of experimental techniques for densely mapping neuronal connectomes [2],[3],[13]. Methods appropriate for quantifying complex structure need not wait for this, however, and can be developed in conjunction with simulations [28]. Moreover, simulation results that make predictions regarding the second question can help inform experimental approaches that combine neuronal connectome mapping with functional analysis, such as reported by [16].

It has mostly been overlooked, however, that the ‘real-world’ complex networks for which existing network science metrics were developed [8],[9] are in several ways dissimilar to networks of neurons and chemical synapses (see Methods). Possibly for this reason, the theoretical network science literature has not yet reported derivation of general principles applicable to understanding how complex synaptic connectivity might affect so-called ‘balanced’ cortical excitation and inhibition (see Methods).

Therefore, the first aim of this paper is to emphasise the need for network models of cortical connectivity (whether hypothesised or based on future neuronal connectome data), and any associated mathematical analysis, to take into account the specific set of properties of neuronal networks described in Methods, such as different classes of nodes and directed edges. A consequence of these properties is that many standard metrics from network science such as overall degree distribution, clustering coefficient and average path length [29] have limited utility if applied to the network in its entirety, unless broken down to describe particular subnetworks. For example, in a network with two neuron classes—excitatory (E) and inhibitory (I)—three subgraphs can be readily identified: (i) the subgraph of E neurons and E
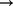
E synapses; (ii) the subgraph of I neurons and I
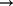
I synapses; (iii) the bipartite subgraph of all neurons but only the E
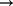
I and I
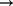
E synapses. Based on the little experimental evidence available, it is likely that each subnetwork exhibits distinct complex structure [30]–[33], in addition to distinct connection densities (see Discussion).

Newly developing network science theory on the topic of undirected multiplex topologies [34]–[36] may be particularly relevant to this topic. Such networks consist of different classes of edges, and it can be interesting to consider separately the topology of each subnetwork that can be formed from all nodes plus edges of just one type. This concept alone, however, is not sufficient for capturing the points raised above. First, there has not, to our knowledge, been any theory developed for directed multiplex networks. Second, a generalisation of the multiplex network concept would be required, where there are multiple node classes as well as multiple edge classes, and only one kind of directed edge can connect nodes of a given class.

The second aim of this paper is to demonstrate the impact on balanced excitation and inhibition of introducing complex clustered network connectivity, and different structural classes of neurons, into simulations of an existing model [23],[37] of cortical activity. This model has realistic neuronal and synaptic dynamics, and exhibits balanced population activity, including so-called ‘sparsely synchronised population oscillations’ [38],[39]. We found that introducing highly clustered network topology, and neurons that can be divided into classes based on their structural connectivity (specifically, their motif participation distributions), can cause bistable population dynamics arising from stochastic bistability in firing rates in one of the classes. This bistability is induced in neurons that receive strong local excitation but only weak non-local inhibition. Neurons in this class exhibit large differences in the distributions of certain two and three synapse network motifs [40] in which they participate, compared with those in other classes, and with a random network. Thus, the global motif distribution of the network is not as good an indicator of dynamics as the distribution of motifs in which individual nodes participate.

## Methods

The sparsely synchronised population oscillations model is a special case of a broader class of cortical network models, in which so-called balance of excitation and inhibition occurs [41]. It has frequently been observed that in order for simulated cortical networks to reproduce experimental observations, the total excitation and inhibition that each neuron receives via spikes from its neighbours needs to be balanced, so that the net resultant synaptic currents lead to membrane potentials that are close to, but below, threshold, and approximately constant over time, apart from small fluctuations that give rise to occasional spikes. A large number of theoretical studies have investigated balanced excitation and inhibition [42]–[51], including recent extensions to the notion of ‘detailed balance’ [52].

We studied the influence of complex network topology and classes of structurally distinct neurons in the context of a ‘balance’ model useful for understanding a particular kind of oscillatory signal recorded from cortical region V1. When awake animals are shown moving images, this region exhibits *in-vivo* local field potentials (LFP) with narrowband oscillations centred in the gamma band (i.e. about 40 Hz) [53]. Empirical data also show that individual neurons in the region are not synchronised, and fire irregularly at an average rate much lower than 40 spikes per second. These findings have been explained mathematically in terms of an effect called ‘sparsely synchronized oscillations,’ which is due to interactions between excitatory positive feedback and inhibitory negative feedback—see [39],[54] for reviews. Simulations also readily exhibit the effect [23],[37],[38],[55]–[57]. Recently we reported briefly [23] that complex structural connectivity can give rise to loss of population oscillations in the specific model of [37], but did not observe bistable dynamics like those reported in the current paper.

Many existing models and simulations of balanced excitation and inhibition, and sparsely synchronised population oscillations, use realistic neuronal membrane and synaptic parameters based on experimental knowledge. The biological realism of the network connectivity model is not yet as well developed, however, and either random or all-to-all networks are typical model choices, since there are few experimental data as to which network topologies actually exist in cortex. This has recently begun to change, and there have now been a number of reports of the influence of ‘non-random’ complex network connectivity on balanced excitation and inhibition in simulations of large networks [19],[20],[22]–[27]. Before that, there had also been several studies into the impact of topology on hyperexcitability [10],[18]. We next describe how realistic complex network topologies with multiple node and edge classes may be introduced into models of such networks.

### Modelling complex networks with multiple classes of neuron and synapse

By definition, a graph consists of a set of 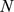 nodes (also called vertices) and 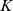 edges, which are defined as connected pairs of nodes. The edges may be directed or undirected. A complex network is a network with connectivity structure that is neither entirely random, nor entirely regular, such as a small-world or scale-free network [14]. Such complex structure has been referred to as ‘non-random’ [58], but rather than imply that connectivity is predictable for arbitrarily selected nodes, this emphasises only that complex networks cannot be generated by making independent random choices (with the same probability) about which edges should exist.

Due to the crucially important fact that some neurons communicate synaptically-excitatory spikes to their neighbours, and others communicate synaptically-inhibitory spikes [59], realistic complex cortical network models require a graph model that includes the following minimal set of properties:

- **Property 1:** nodes represent individual neurons;
- **Property 2:** edges are directed and (usually) represent chemical synapses;
- **Property 3:** there are at least two distinct node classes: excitatory (E) & inhibitory (I);
- **Property 4:** there are at least four distinct directed edge classes: E
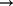
E, E
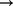
I, I
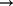
E and I
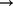
I;
- **Property 5:** average connection densities may be different for each edge class (see Discussion).

This set of properties is general enough to be applicable to other kinds of neuron-to-neuron communication besides chemical synapses, such as the two-way gap junctions observed in both *C. elegans* [6], and between cortical inhibitory neurons [60], and autapses (self-synaptic connections) [17],[61].

Since topological structure may differ in the same network depending on which neuron classes are considered, this means quantifying complex structure can only be meaningfully achieved if the network is decomposed into subnetworks.

Besides the excitatory and inhibitory classes, historically other divisions of cortical neurons into classes have been defined based on physiology and anatomy—we suggest that these be referred to as **physiologically-defined neuron classes**. It has recently been argued that neuron-to-neuron connectivity is likely to be a more informative basis upon which to partition neurons into classes [2]. We refer to such classes as **structurally-defined neuron classes**, and next introduce mathematical notation suitable for describing either kind of classification.

### Notation for networks with different node and edge classes

Any network with 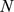 nodes that has no more than a single edge in the same direction between nodes can be represented by its 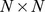 adjacency matrix, 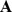 [8]. For an unweighted and directed network, as considered exclusively in this paper, the elements of this matrix, 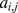, are binary, such that 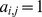 when a directed edge originates at node 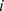 and ends at node 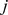 (we denote this as 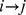) and 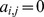 otherwise, where 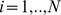 and 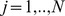. For such an undirected network 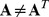 in general. In this paper we also exclude all edges from a node to itself, so that 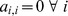.

Networks are often characterised by their degree-distributions [14]. Degree is defined for a single node as the number of edges in which it participates. For a directed network, the in-degree of node 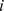 is the number of directed edges that end at node 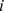. We denote this as 
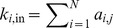
. The out-degree of node 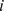 is the number of directed edges that originate at node 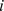. We denote this as 
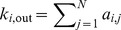
. The mean in-degree for the network is 
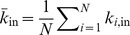
. The mean out-degree for the network is 
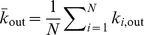
. We write the in-degree distribution as 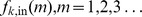 and the out-degree distribution as 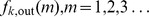.

We introduce general notation applicable for an arbitrary number, 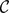, of node classes and 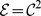 edge classes. Of these, we define 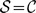 as the number of edge classes whose edges start and end in the same class of node and 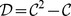 as the number of edge classes whose edges start in one node class and ends in another. We denote each node class with the label 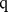 and the number of nodes in each class as 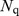, so that 
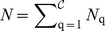
.

There is an in-degree distribution for each pair of node classes, and an out-degree distribution for each pair of node classes, for a total of 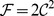 degree distributions. We denote the in-degree to the 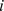
–th node of type 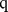, from nodes of type 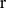, as 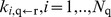. We denote the out-degree from the 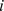
–th node of type 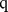 to nodes of type 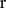 as 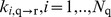. Thus, the direction of the arrow in the subscript indicates in or out degree.

Similarly, the mean in-degree for nodes of type 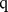 from nodes of type 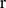 is denoted as 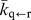 and the mean out degree for nodes of type 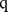 to nodes of type 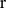 is 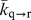


For example, if there is a distinction between only two classes of nodes, such as excitatory and inhibitory, then 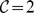, 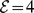 and 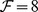. Two of the eight degree distributions are associated with the EE subnetwork, two with the II subnetwork, and four are associated with the bipartite EI/IE subnetwork. When we consider these two node classes only, we introduce class labels 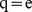 and 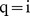. Elsewhere, we consider six classes, and the labels are introduced below.

### Null hypothesis random network: Constant in-degrees for all classes

In order to compare the effect of changes in topology, or the influence of complex non-random features in empirical networks, it is necessary to compare results with those from an appropriately chosen null-hypothesis network [5]. Since we are motivated by the question of how network activity depends on topology changes with respect to a random network of size 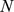, a random network is the obvious choice for the null hypothesis network. Additionally, we chose to impose one further constraint on our null hypothesis random network, which is that the in-degree to a node class from each edge class is constant for all nodes in the class. The reason for this is as follows.

Intuitively, a neuron in a network will be more likely to produce action potentials as the ratio of its incoming excitatory synapses to incoming inhibitory synapses increases. This is plausibly a false intuition. For example, complex feedback pathways and quirks of spike timing caused by variable delays, may render it false. Nonetheless, we ensure this ratio is not the cause of any changes in network dynamics by forcing all neurons in all networks to have the same in-degree from each class of neuron. For similar reasons, the same network topology was studied in [62].

We therefore introduce (even valued) 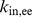 such that 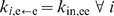 and (even valued) 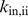 such that 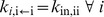. Similarly, we define (even valued) 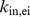 such that 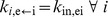 and (even valued) 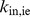 such that 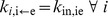.

For all networks considered in this paper, we impose the constraint that 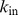 is identical for all neurons, and that 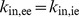 and 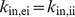. Consequently, we have also that 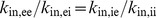.

We label the resultant control or null-hypothesis network as 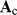.

### Construction of three ‘non-random’ network topologies

We aimed to create networks with constant in-degrees, such that subnetworks of neurons were highly clustered, but there were short paths between any pair of nodes in the network.

One way to ensure a highly clustered network is to create a regular lattice or regular ring lattice topology. Although undirected ring lattice networks are prevalent in network science due to their role in the seminal small-world network model of [63], directed ring lattice networks are less frequently studied, with some exceptions [19],[23],[64]. Here we consider a version of a directed ring lattice, and its conversion to a directed small-world network, as first introduced in [23].

#### Forwards-backwards excitatory and inhibitory ring lattices

We assume 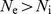, that 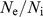 is integer, and that 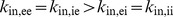, so that 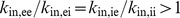.

**Step 1:** Create a forwards-backwards ring lattice directed network for E-nodes, and store this in an 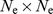 square matrix 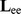:

For all 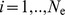, set 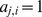 for 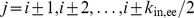 (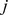 indices are modulo 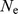).

**Step 2:** Create a forwards-backwards ring lattice directed network for I-nodes and store this in an 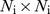 square matrix 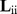:

For all 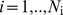, set 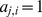 for 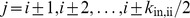 (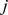 indices are modulo 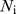).

**Step 3:** Create 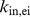 edges to each E-node from I-nodes. Store these in an 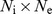 non-square matrix 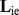. We want to resemble the forwards-backwards ring lattice. Therefore we construct the network such that E nodes with adjacent indices receive input from the same I node. Since the E ring lattice is bigger than the I ring lattice, a set of E nodes with contiguous indices can receive connections from identical pools of I nodes:

For all 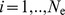, set 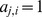 for 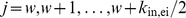 and 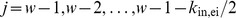 (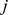 indices are modulo 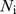), where 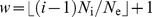.

**Step 4:** Create 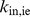 edges to each I-node from E-nodes. Store these in an 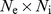 non-square matrix 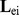. We want to resemble the forwards-backwards ring lattice. Therefore we construct the network such that I nodes with adjacent indices receive input from the same E node. Like the forwards-backwards ring lattice, we ensure that E nodes with adjacent indices connect to I nodes with adjacent indices:

For all 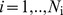, calculate 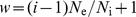 and set 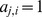 for 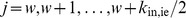 and 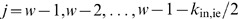 (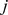 indices are modulo 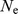).

**Step 5:** Compose the overall lattice network's 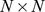 adjacency matrix as
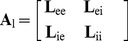
(1)


Note that this network is highly clustered, which can be readily quantified using the directed clustering coefficient metric of Fagiolo [65], as shown in [23].

#### Directed small-world network with structurally-defined neuron classes

We begin with the directed ring lattice network described above, and randomly choose some edges to be removed and replaced by other edges. Our rewiring algorithm ensures the in-degree of cells is unchanged, by selecting edges for rewiring, and then only rewiring the origin node for that edge, while ensuring the new node of origin is of the same class as the original node of origin. Consequently, all neurons receive exactly 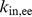 excitatory synapses and 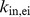 inhibitory synapses, both before and after rewiring.

In the classical Watts-Strogatz small-world network [63], rewiring proceeds by looping over every edge in a deterministic ring lattice, and rewiring it such that with probability 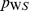, one of its nodes is replaced by another randomly selected node.

We considered several rewiring methods, including one where all edges are considered independently. The most interesting results, however, were obtained for a method in which, rather than considering all edges for rewiring, we chose nodes with probability 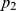 to have the originating node for all its incoming inhibitory edges replaced with randomly selected inhibitory nodes. We label the chosen nodes as *Class 2* nodes. Crucially, we ensured that the new originating node for each incoming edge to the selected nodes was not connected to the selected node in the original lattice network. We found different and less interesting outcomes when it was possible for original edges to be selected for re-inclusion. We then randomly chose nodes from those not chosen to become Class 2 nodes, with probability 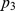, to have all their incoming edges rewired at the originating end. We label the chosen nodes as *Class 3* nodes. Nodes that were not chosen in either case are labelled as *Class 1* nodes.

The resulting rewired network, which we label as 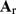, can be expressed in terms of the following decomposition. We begin by introducing ‘mask’ matrices, 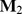 and 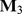 whose columns are equal to unity for all *Class 2* and *Class 3* nodes respectively, and zero otherwise. These mask matrices are constructed such that each row is identical and given by length 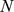 random vectors, 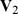 and 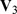, that each contain 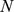 independent outcomes from binary random variables that are unity with probabilities 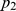 and 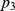 respectively. The entries that are unity are the indices of nodes that are selected for rewiring.

The resulting adjacency matrix for the rewired network can be expressed as
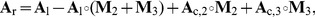
(2)where 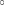 indicates Hadamard (element wise) multiplication. The matrix 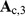 is a directed random graph of the same nature as our null-hypothesis network, 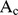, that is, it is random and constrained only by the fact that all in and out-degrees from and to each node type (**e** and **i**) are constant, since there are no other constraints on from where *Class 3* nodes receive their randomly selected inputs. The matrix 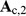, however, is additionally constrained such that its elements must be zero when corresponding elements of 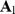 are unity. Note that the first two terms on the left hand side of Eqn. (2) describe connections to *Class 1* nodes, the third term describes connections to *Class 2* nodes and the final term describes connections to *Class 3* nodes. By the definition of the classes, no similar general statement can be made about connections from nodes of different classes.

Because both excitatory neurons and inhibitory neurons can be rewired in either way, our rewiring process creates 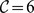 **structurally-defined neuron classes**. These 6 classes, the labels we give them, and the mean number of nodes in each class, are summarized in Table 1. With this many classes, there are 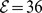 edge classes. Our algorithm ensures that the mean in-degree to a node in class 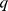 from nodes in class 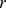 is exactly 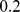 times the number of nodes in class 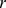. Similarly, the mean out-degree from a node in class 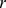 to nodes in class 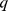 is exactly 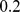 times the number of nodes in class 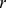.

**Table 1:** List of structurally-defined neuron classes in rewired ring lattice, and size of each class for the example of 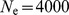, 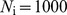, 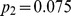, 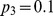.

Class label, q	Mean node count
E1	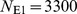
E2	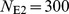
E3	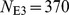
I1	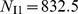
I2	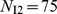
I3	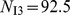

We define Class 1 neurons as the union of classes E1 and I1 (E and I neurons with no synapses rewired), Class 2 neurons as the union of classes E2 and I2 (E and I neurons with all incoming inhibitory synapses rewired at originating end) and Class 3 neurons as the union of classes E3 and I3 (E and I neurons with all incoming excitatory and inhibitory synapses rewired at originating end).

#### Embedded modular excitatory neworks

The final network we studied was a version of the network described by [27], in which subnetworks or modules of highly clustered excitatory neurons are present. Two versions of such networks are described by [27], but we studied only the homogeneous version, in which all excitatory neurons belong to exactly one non-overlapping module. The network is characterised by several parameters: the number of neurons in each excitatory module, 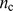, the probability of an edge within a module existing, 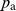, the probability of an edge that begins in one module and ends in another existing, 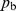, the probability that an edge involving an inhibitory neurons exists, 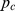, and the overall mean fraction of E
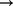
E connections, 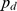. The number of excitatory neuron clusters is 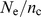. The E
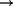
E connectivity within a module is controlled by the ratio 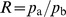, subject to the requirement that 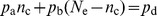. This enables 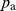 and 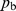 to be derived for arbitrary 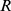 such that 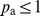. We label the adjacency matrix of resulting networks of this category as 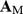.

### Neuron and synapse models and parameters

For the dynamics of neuronal membrane potentials and synaptic currents, and synaptic communication between neurons, we used the same model described in [23],[37], except that we modified it in one particular way that improves its biophysical realism, as well as ensuring more stable dynamics. Specifically, we replaced the current synapse model of [23],[37] with a conductance synapse model (see, e.g. [55] for the differences, and discussion in the context of balanced cortical activity).

In our model, the state of each node is given by the membrane potential, 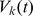, of each neuron, 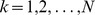:
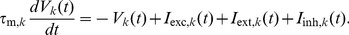
(3)At 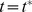, if 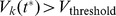, then 
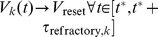
, and neuron 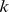 communicates a spike on all its outward edges. The variables 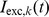, 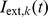, and 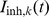 represent membrane currents resulting from input into nodes, either from other nodes (exc,inh), or from external to the network (ext).

Communication between nodes via chemical synapses is by spike propagation (a directed process). The external input is comprised of spikes that arrive at each neuron according to independent Poisson random variables with mean 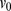 spikes per ms, representing input from many neurons in the thalamus. This is a slight modification of the way that external spikes are generated in [23],[37]. Here we did not make the Poisson rate, 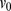, a noisy rate, but simply generated spikes independently for each neuron according to a constant Poisson rate.

Spikes are ‘sent’ from node 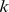 to all its neighbours in the network when 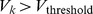, and when they arrive at a node, after a delay, 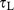, they begin to elicit a synaptic current of type 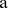 where 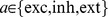. The synaptic current in node 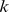 due to input spikes arriving at times 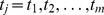 is
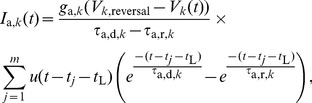
(4)where 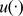 is the Heaviside unit step function. This model of synaptic response is known as the difference of exponentials model, and is characterised by distinct rise times, 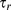 and decay times, 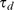, and in general we use different values depending on the synapse class, as in [23],[37],[38].

Note that unlike in [23],[37], the current is dependent on the membrane potential, and the exponential rise and fall times characterise the change in conductance as a function of time, rather than of current.

The parameter values we used are listed in Tables 2 and 3, and are the same as those used in [23],[37], except for the synaptic efficacies, which we modified to be applicable to the conductance model, and the reversal potentials, which were not used in those papers.

**Table 2:** List of parameter values for neurons in the model.

Nodal Parameters	Node type	Notation	Value
Membrane time constant	E	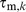	20 ms
	I	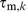	10 ms
Reversal potential	E	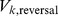	70 mV
	I	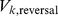	0 mV
Refractory time	E	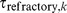	2 ms
	I	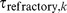	1 ms
Threshold potential	E and I	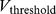	18 mV
Reset potential	E and I	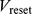	11 mV

All parameter values are taken from [37], except for the reversal potentials. Neurons in the model are indexed such that 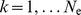 when 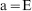 or 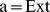 and 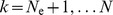 when 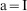.

**Table 3:** List of parameters for synapses in the model.

Edge (Synaptic) Parameters	Edge type	Notation	Value
Onset latency	All	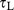	2 ms
Synaptic rise time	Ext & E to E	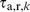	0.4 ms
	I to E	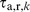	0.25 ms
	Ext & E to I	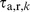	0.2 ms
	I to I	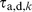	0.25 ms
Synaptic decay time	Ext & E to E	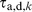	2 ms
	I to E	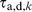	5 ms
	Ext & E to I	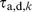	1 ms
	I to I	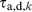	5 ms
Synaptic efficacy	E to E	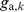	0.028
	E to I	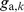	0.011
	I to E	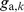	0.113
	I to I	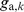	0.180
	Ext to E	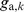	0.008
	Ext to I	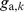	0.014

All parameter values taken from [37], except for the synaptic efficacies, which were modified to be applicable to the conductance synapse model. Neurons in the model are indexed such that 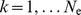 when 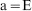 or 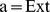 and 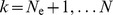 when 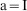.

### Local field potential model

As proposed in [37], we model the LFP of the network as proportional to the sum of the absolute values of incoming currents to excitatory neurons,
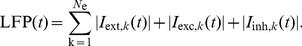
(5)Note that here since we have conductance synapses, each current, and therefore the LFP, depends on each neuron's membrane potential, unlike the current synapse model of [37].

### Balance analysis

We devised two different tests for balanced excitation and inhibition. We refer to networks classified as being unbalanced as being in an ‘*upstate*’ (due to higher than average firing in some neurons in this state), and otherwise refer to the network as being in a ‘*downstate*’. The first test quantified balance for the overall network. The second enabled individual neurons to be classified as being in one of three states.

#### Method 1: overall network

In order to generate large sample-sizes efficiently, simulated LFP data obtained using Eqn. (5) were used, thus avoiding the need to store large amounts of data from individual neurons. We used a two-pass algorithm applied to simulated LFP data. First, the simulation data were smoothed with a 100-ms duration uniform-kernel window. A baseline window was determined as the window whose mean was the smallest out of all 100-ms windows. Preliminary upstates were identified as those successive samples where the 100-ms window mean was greater than 3 baseline-window standard deviations above the baseline-window mean. Finally, the second pass employed a 5-ms duration uniform-kernel window based on the geometric mean (as the sample LFP distributions were highly positively skewed). The beginnings and endings of the remaining preliminary upstates were adjusted using the same criterion but comparing the baseline mean to the geometric means of the 5-ms windows. Finally, we computed the geometric mean LFPs and the durations of the final set of upstates and downstates.

#### Method 2: individual neurons

For the purposes of identifying the mechanisms responsible for transitions from downstates to upstates and upstates to downstates, we also devised a method for classifying each neuron at any point in time during a simulation as either being either in a neuron ‘*high*’, ‘*neutral*’ or ‘*low’* state. As shown in Results, for the rewired ring lattice network a network upstate generally coincides with many neurons in all three states, while the network downstate coincides with the majority of neurons being in the neutral state. A neuron was classified as being in the low state when its membrane potential was significantly lower than the mean membrane potential in a random network. A neuron was defined as being in the high state when it fired significantly more frequently in a recent interval than in a random network. A neutral state was the case of neither the high or low state. Based on observations from a random network, we used a threshold of 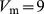 mV to determine the low state, and a threshold of 1 spike in the last 15 ms to determine the high state.

### Complex network metrics: participatory motifs

The number of reciprocal connections for each node (i.e. when a neuron is synaptically connected in both directions with another neuron) is determined by calculating 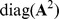, where the 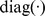 operator extracts the 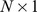 vector of diagonal entries from a square matrix of size 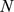.

Counts of the number of particular types of three-edge motifs in which each node participates can be found by calculating 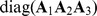, where 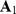, 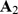 and 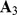 are three tailored subnetworks of 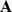.

For example, for feedforward convergent motifs that end in node 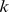 and involve an excitatory edge, 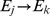, an excitatory edge 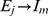 and an inhibitory edge, 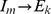, we have
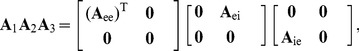
(6)where 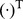 is the matrix transpose and the submatrices 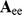, 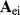 and 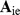 are submatrices describing subnetworks of the overall adjacency matrix.

For cyclic motifs that start in node 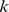 and involve an excitatory edge, 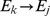, an excitatory edge 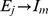 and an inhibitory edge, 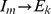, we have 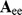 in the first term rather than 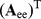.

## Results

Each neuron's activity was simulated in a network of 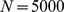 neurons (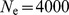 excitatory, 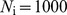 inhibitory) for durations typically of 5.1 seconds (see figure captions for exceptions), with a simulation step size of 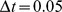 ms, and an external Poisson spike rate of 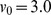 spikes per millisecond. The input spikes were generated independently for each neuron. Both the spike-train realisations, and network realisations were generated multiple times, to ensure that results were robust to stochastic changes in both topology and external drive. Since all variables were initialised to zero, the first 100 ms were discarded for most figures, in order to avoid biasing results with transient effects.

Four network topologies were compared, each with fixed in-degree (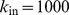 for all neurons; 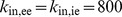 and 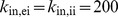):

1. random, with fixed in-degree;
2. deterministic forwards-backwards ring-lattice;
3. rewired forwards-backwards ring-lattice with 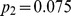 and 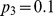, except where otherwise indicated (see Methods and Table 1).
4. embedded modular excitatory networks with 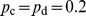, 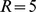, and 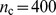, which leads to 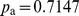 and 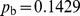 (see Methods).

For the embedded modular excitatory networks, we found it necessary for two reasons to choose different parameters than in [27], where 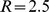, 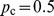, 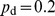, 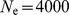 (as studied here) and 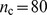 excitatory neurons in each module, so that there were 50 modules. First, we chose 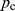 to be smaller than in [27], in order for consistency with the other networks we studied. Second, we found that both larger modules (and therefore fewer of them), and higher E
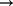
E connectivity within those modules, were necessary to induce network behaviour significantly different from the null-hypothesis random network.

### Testing for balanced excitation and inhibition

Balanced excitation and inhibition is often quantified in theoretical work in terms of the variance of the membrane potential over time in each neuron in a network. We observed that some network topologies gave rise to non-stationary membrane potentials, however, and therefore decided that it would be more revealing to study how the spike rates for each neuron varied over time and with respect to topology.

**Figure 1:**
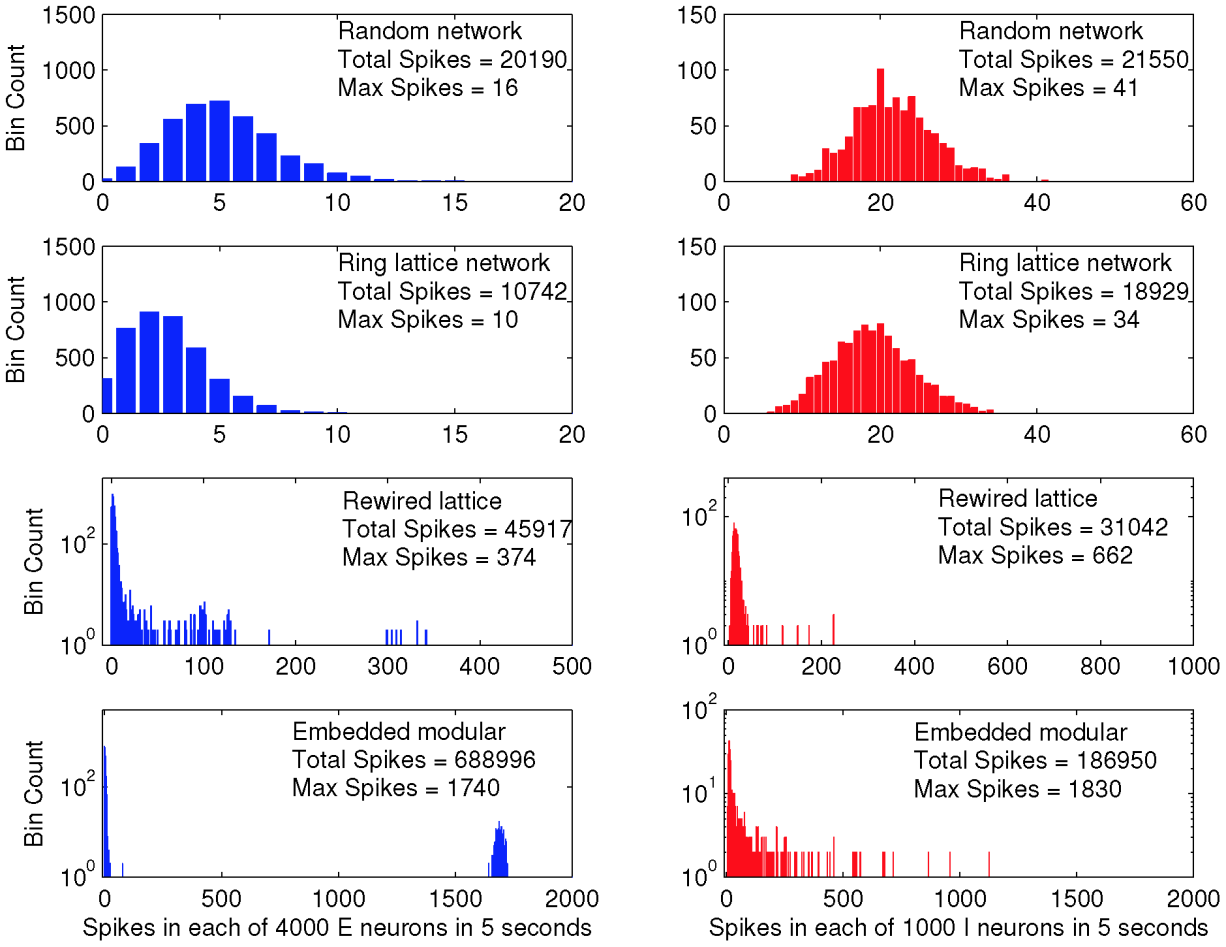
Spike counts in a 5s simulation indicate loss of balance for two complex network topologies. Each subfigure shows the frequency of neuron spike counts over the 5s simulation for four distinct network topologies. Data for excitatory neurons are shown in the left column and data for inhibitory neurons in the right column. The first row shows data for the random network, the second row the lattice network, the third row the rewired lattice network with 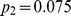 and 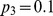, and the fourth row the network with embedded modular excitatory subnetworks. The rewired lattice and modular networks each have numerous neurons that spike much more frequently than those in the random or lattice networks, and also produce many more spikes in total.

Figure 1 shows histograms of the total number of spikes for each neuron in a single simulation run of five seconds, for single realisations of each of the four network topologies, with the 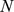 random input spike trains required as input to each neuron in the model repeated in each topology. In the random and lattice networks, the mean and maximum spike rates are consistent with previous simulations of the overall model [37], and with experimental observations of balanced networks. In particular, the mean spike rate (approximately 1 spike per second for E neurons and approximately 4 spikes per second for I neurons) is significantly less than the gamma frequency oscillations at over 40 Hz for both kinds of neurons, previously observed in both these networks [23].

**Figure 2:**
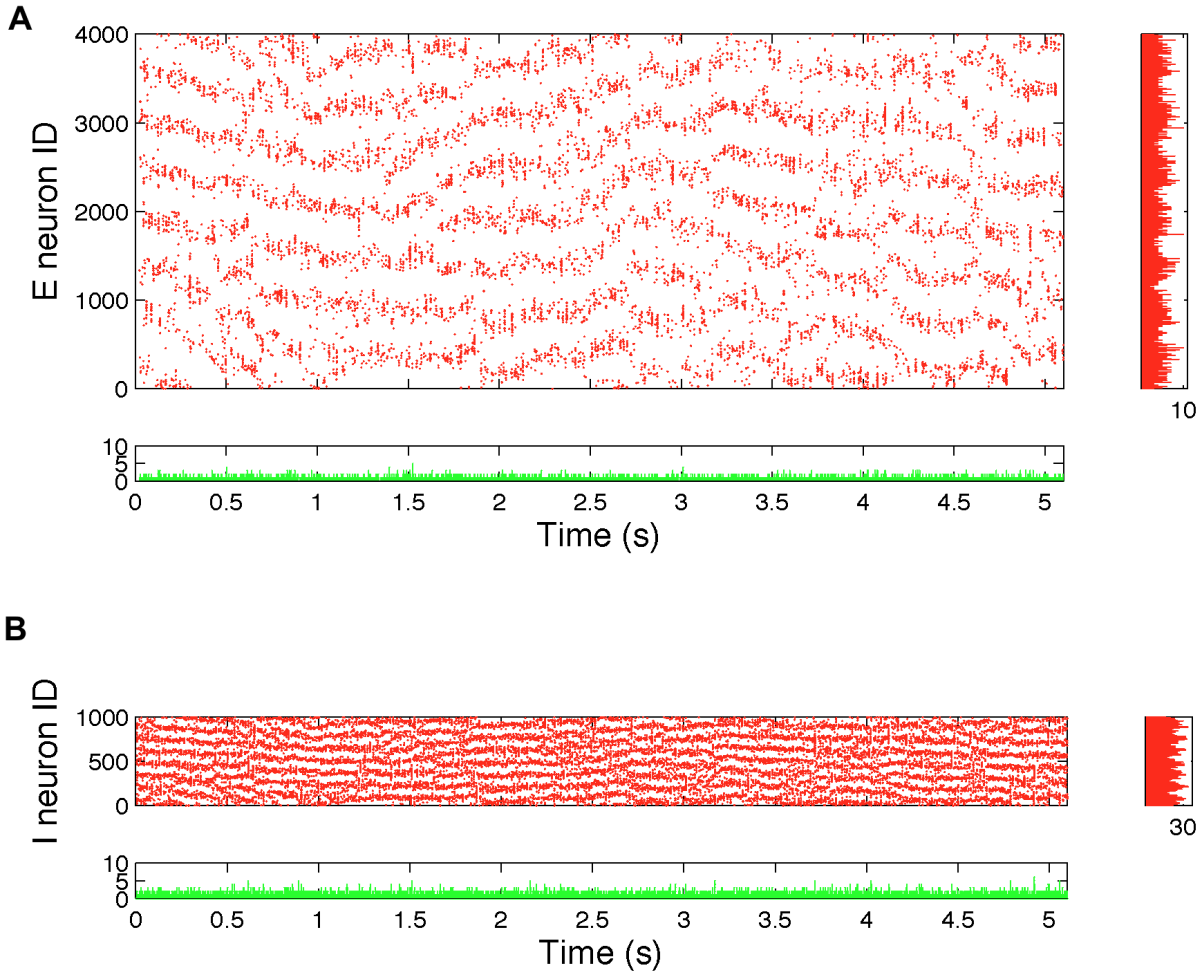
Raster plots of simulated spikes for the deterministic ring lattice exhibit spatial correlations. This figure shows each spike from a single 5.1s simulation with the deterministic ring lattice network. Data for excitatory neurons are shown in A and data for inhibitory neurons in B. Shown underneath each raster plot is a bar plot of the total number of spikes in each simulation time step (0.05 ms). Shown to the right of each raster plot are bar plots of the total number of spikes in each neuron over the entire 5.1 seconds. Although the overall spike rates suggest balanced activity, the spatial correlation apparent in the raster plots are not observed in a random network (see Figure S1). These spatial correlations may help initiate upstates in the network consisting of a rewired ring-lattice (see Figure 3).

However, a raster plot of all spikes in the deterministic ring lattice (Figure 2) show a spatial correlation structure, with partial wave propagation and local clusters of higher activity. This is despite the fact that by design, every neuron projects equally forwards and backwards around the E and I ring lattices, and the overall average spike rates are similar to the random network. There is no such structure for the random network (See Figure S1). Consequently, it is debatable whether the deterministic ring lattice should be labelled as a balanced network.

**Figure 3:**
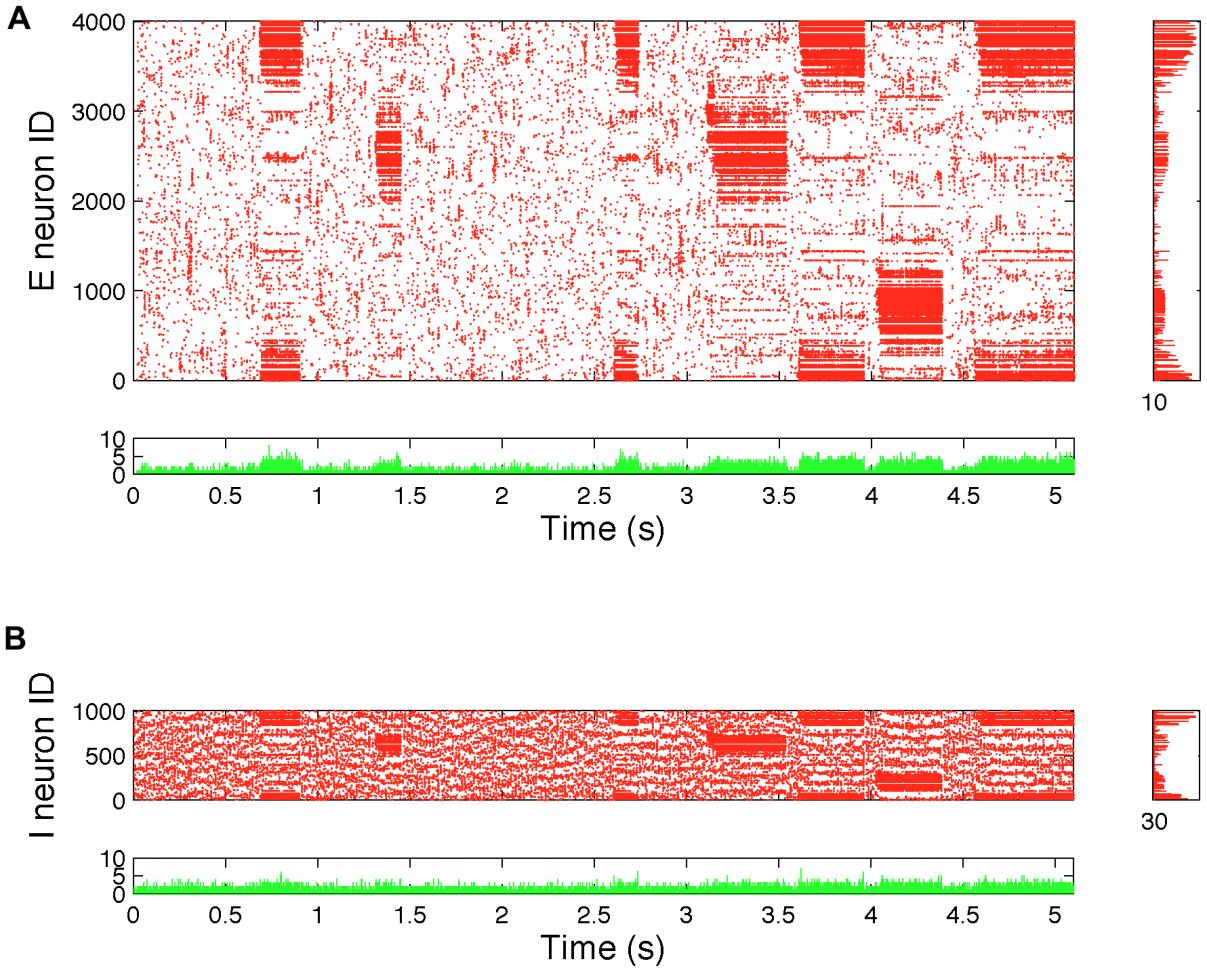
Raster plots for the rewired ring-lattice network show loss of balance due to ‘upstates’ and ‘downstates’. This figure shows each spike from a single 5.1s simulation for the rewired ring-lattice network with 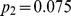 and 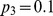. Data for excitatory neurons are shown in A and data for inhibitory neurons in B. Shown underneath each raster plot is a bar plot of the total number of spikes in each simulation time step (0.05 ms). Shown to the right of each raster plot are bar plots of the total number of spikes in each neuron over the entire 5.1 seconds. This data shows that the high spiking in some neurons apparent in Figure 1 is not homogeneous over time, but results from epochs of much higher than average firing.

Nonetheless, in contrast to the random network and the ring lattice, both the randomly rewired lattice, and the network with embedded modular excitatory subnetworks show spike count histograms highly inconsistent with balanced excitation and inhibition, since although some neurons do have firing rates similar to those in the random network, there are neurons that have much higher firing rates, despite their identical intrinsic dynamics and in-degrees. The imbalance is more readily apparent in Figure 3, which show a raster plots for all neurons in the rewired ring lattice, from a simulation of duration 5.1 seconds (see also Figure S2 for raster plots of data from the embedded modular excitatory network). This figure shows that there are epochs of time when different subsets of neurons fire at much higher rates than usual. We term these epochs ‘upstates’ and contrast them with epochs when no particular subset of neurons is firing at a higher than average rate for any sustained period, which we term ‘downstates’. More detailed analysis of this network bistability and its origin follows.

### The randomly rewired ring-lattice can robustly exhibit unbalanced bistable dynamics

For the case of 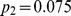 and 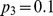, it was found that bistable states are generated robustly for different realisations of the rewired lattice network and different realisations of input spike trains to neurons in the same network realisation. Figures S3, S4, S5, S6, S7 show plots of the population local field potential (LFP) for the model proposed by [37] (see Methods). The model LFP data are shown for five independent runs, for the same single realisation of the randomly rewired lattice network in comparison with data for five independent runs for the same single realisation of the deterministic ring lattice network. We plotted these data instead of spike rates, as we have found them to be an accurate predictor of changes in firing rate. It is clear that the ring lattice network never goes into the highly active state, but the LFP in the rewired network transiently switches back and forth between upstates and downstates.

**Figure 4:**
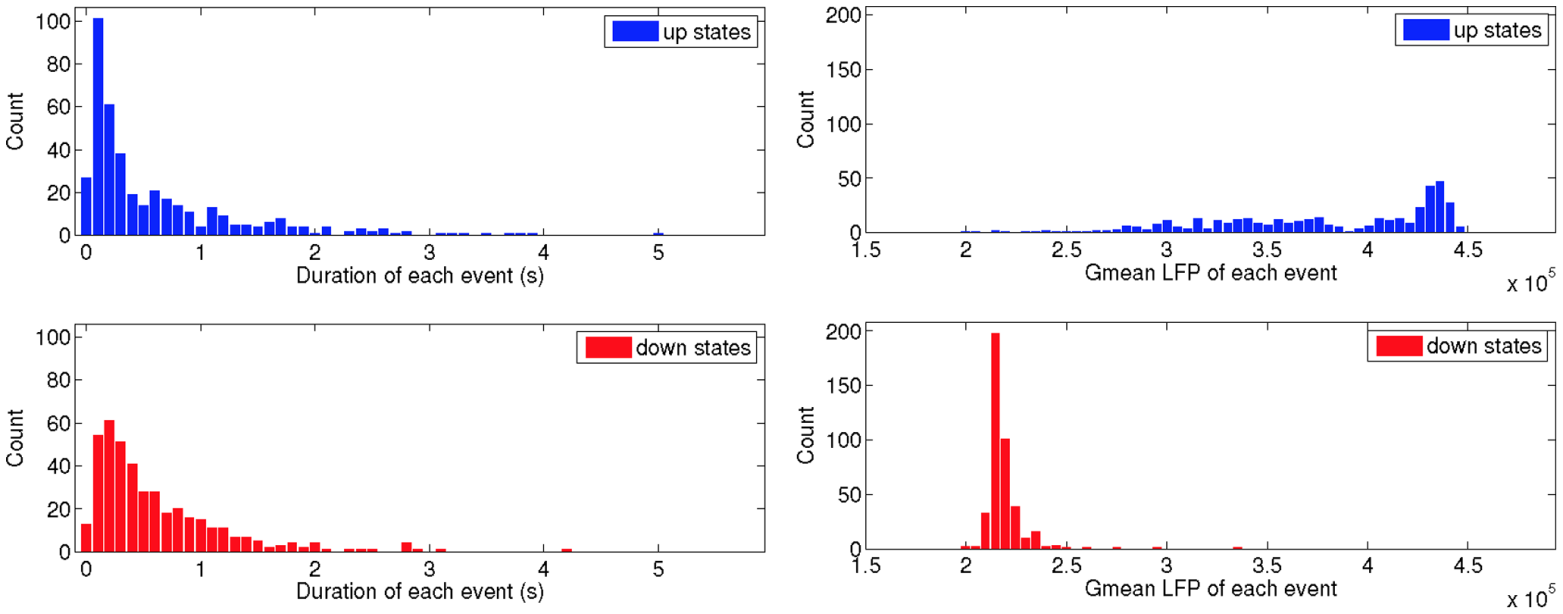
Bistable switching between downstates and upstates occurs robustly in the rewired ring-lattice network. This figure shows histograms of the lengths and geometric means of upstates and downstates in the rewired ring-lattice network, with 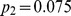 and 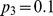, from a simulation of overall duration 500 seconds. A total of 411 switches from down to up and up to down were detected during this time. Plots in the left column show histograms of the durations of the up and downstates, while those in the right column show histograms of the mean LFP during each up and downstate. Given that the longest up and downstates are less than 4.5 seconds, the network clearly robustly switches between up and downstates over a long time period. The mean duration of the downstates was 0.60 seconds, and the mean duration of the upstates was 0.62 seconds, and thus the mean frequency of the bistable state is of the order of one hertz.

In order to quantify variations in the duration of upstates and downstates, and the central tendencies of the LFP during those states, we simulated 500 seconds of neuron activity for a single realisation of the rewired ring lattice. During this period, we detected 411 switches from downstates to upstates, and vice versa. We calculated the duration of each state event, and the geometric mean LFP during each state event (see Methods). Figure 4 shows histograms of the durations of each event, and the geometric mean LFPs of each event.

These data clearly show that the bistable network activity robustly switches between downstates and upstates. The mean duration of the downstates was 0.60 seconds, and the mean duration of the upstates was 0.62 seconds.

Clearly, although there is some dependence on network topology in terms of variance of the population activity, jumps between two clearly demarcated states occur in every plot shown in the Figures S3, S4, S5, S6, S7, and thus we conclude that the bistable behaviour is robust in the sense that it continues indefinitely over time, and for different realisations of the network and/or stochastic external spike inputs.

### Different topological classes induce network bistability

**Figure 5:**
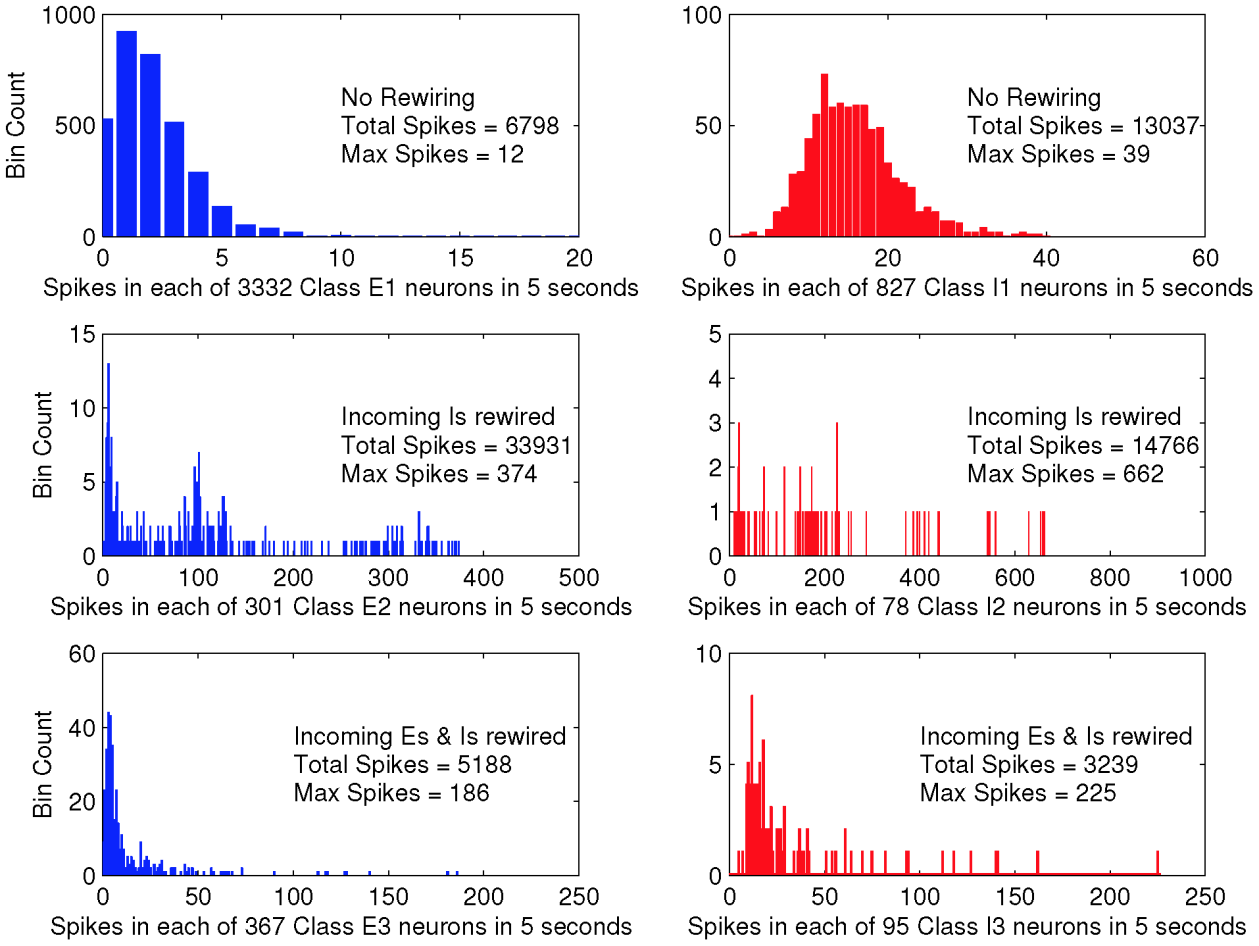
Class 2 neurons dominate firing in the upstates of the rewired ring-lattice network. This figure shows the frequency of spike counts for different structurally-defined neuron classes in the rewired ring lattice, with 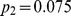 and 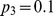. Data for excitatory neurons are shown in the left column and data for inhibitory neurons in the right column. The first row shows data for neurons that were not rewired (Class 1); the second row shows data for neurons rewired only in terms of their incoming inhibition (Class 2); the third row shows data for neurons that had both incoming excitation and inhibition rewired (Class 3). The simulation for each network lasted for 5.1s, but the first 100 ms were discarded. Clearly it is the Class 2 neurons that produce the most spikes, but some Class 3 neurons also fire much more frequently than any Class 1 neurons.

In order to determine how random changes to connectivity in the ring lattice affected the total number of spikes in neurons in the rewired lattice network, we examined the spike rates for neurons in each of the structurally-defined neuron classes (defined in Methods). Figure 5 shows spike count histograms obtained from the data for the randomly rewired ring lattice shown in Figure 1(C), broken down by class. These data show that the main origin of neurons that fire at much higher rates than those in the random network or deterministic ring lattice network are Classes E2 and I2, i.e. those that have had only their incoming inhibitory connections rewired at the origin.

**Figure 6:**
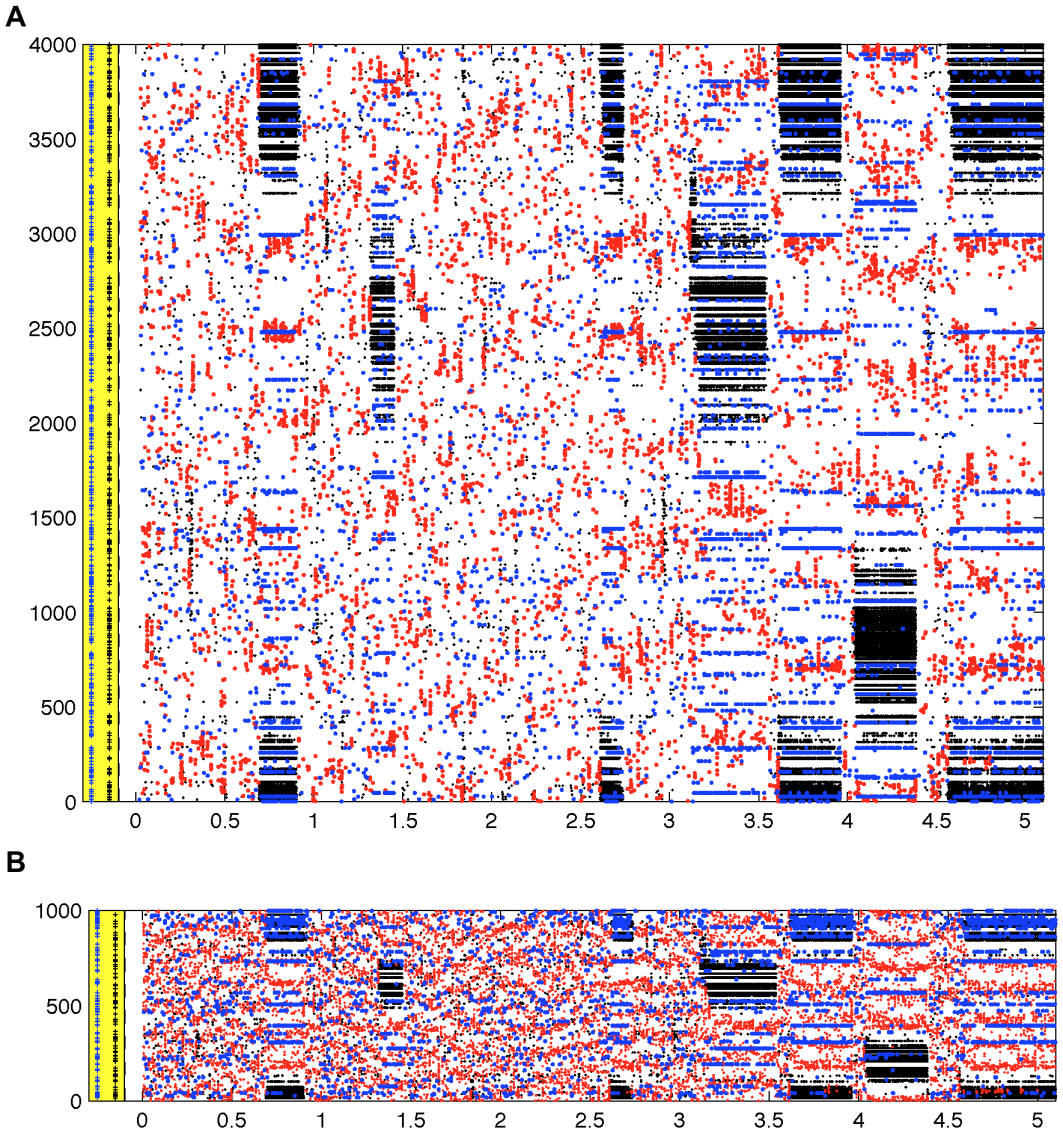
Different structurally-defined neuron classes in the rewired ring-lattice network exhibit different spatial correlations. This figure shows each spike from a single 5.1s simulation with the rewired ring-lattice network, with 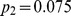 and 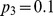, where data for excitatory neurons are shown in A and data for inhibitory neurons in B. Red dots are spikes in neurons that were not rewired (Class 1); black dots are spikes in neurons that had incoming inhibition only rewired (Class 2); blue dots are spikes in neurons that had all incoming synapses rewired (Class 3). The plus marks in the yellow area to the left of the spike data indicate the indices of all Class 2 neurons (black) and all Class 3 neurons (blue). It is clear that during upstates the Class 2 neurons that participate are likely to be spatially close together with respect to the ring-lattice topology. However, Class 3 neurons that participate appear to do so as isolated individual cells.

In order to clarify the source of the imbalance, Figure 6 shows a raster plot where spikes from neurons in each class are shown in different colours. This figure shows that clusters of nearby Class E2 and I2 neurons transiently (on a time scale on the order of a second) fire at high rates, before eventually turning quiescent again. We can state that these neurons are ‘nearby’ each other, because the original network was a ring lattice in which neurons having adjacent indexes determines that those neurons are connected. The cluster of highly active excitatory neurons corresponds to the same position in the original ring lattice as that of the highly active inhibitory neurons.

**Figure 7:**
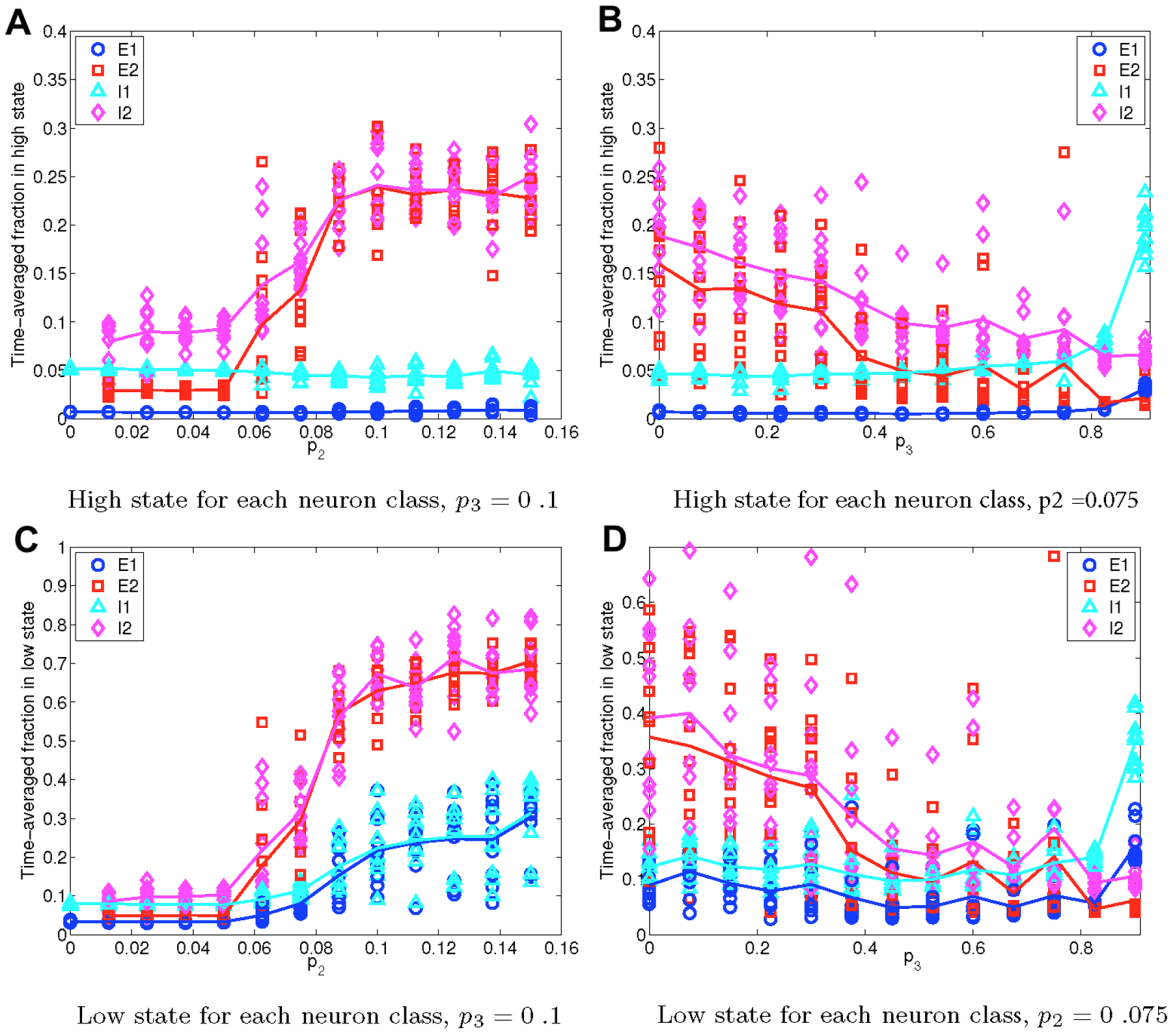
Bistable network activity is observed for 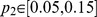, approximately, and is mainly due to high-state Class 2 neurons. Each data point shows the the time-averaged fraction of neurons in each class that was classified as being in the neuron high state (A and B), or neuron low state (C and D) for 10 different realisations of the rewired ring lattice network, as the rewiring probabilities were changed. Solid lines show means from the 10 realisations. Subfigure A shows that increasing 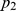 from zero leads to a sigmoidal increase in likelihood for Class 2 neurons being in the high state, with saturation at about 25% at 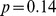. Subfigures B and D show that increasing 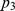, with 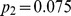, leads to decreasing numbers of Class 2 neurons in the high and low states. Subfigure C shows the fraction of Class 1 and Class 2 neurons in the low state increases as 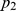 increases with 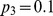, consistent with the explanation in the text regarding high state Class I2 neurons depressing nearby Class 1 neurons with their strong inhibition.

The observation that Class E2 and I2 neurons are those that are most active during network upstates is confirmed by Figure 7, which shows that at 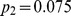 and 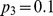 (as considered in all above results), the fraction of E2 and I2 neurons in the neuron high state is, on average much higher than that of Class E1 and Class I1 neurons.

**Figure 8:**
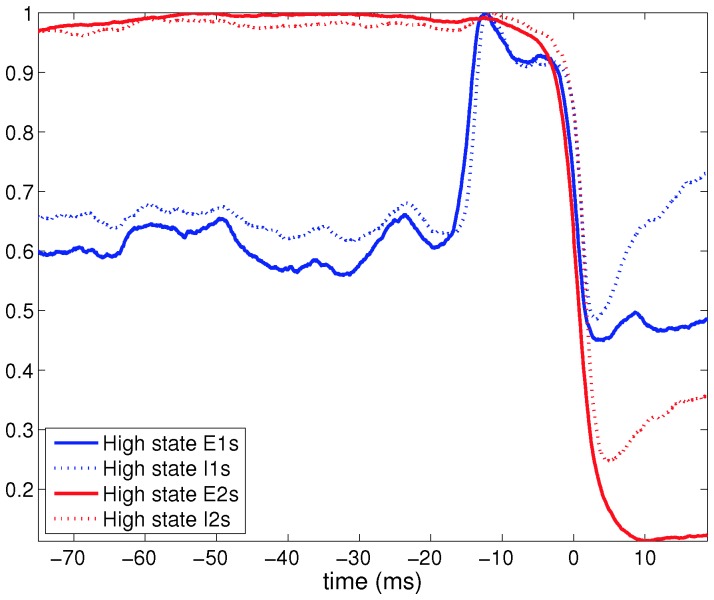
Transitions from upstates to downstates occur following higher than average excitation of E1 and I1 neurons. In the text, the transition from network upstates to network downstates is explained in terms of higher than average non-local inhibition to high state neurons leading to termination of the network upstate. This figure shows simulation data from the rewired ring lattice (with 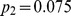 and 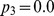). Within a single simulation of duration 250 seconds, 139 up-to-down transition events with at least 75 ms between network upstates were detected. The average number of high state neurons of each kind were calculated for 75 ms before and 20 ms after the transition, and are shown on the figure after normalising by the maximum average number within the time window. The data shows that termination of the upstate (indicated by a rapid drop in the number of E2 neurons in the neuron high state) is preceded by an increase in the number of E1 and I1 neurons in the neuron high state, as described in the text.

In Discussion, we describe a mechanism for transitions between network upstates and downstates. This mechanism is supported by data shown in Figure 8, which shows that transitions from network upstates to downstates (as quantified by the number of Class E2 neurons in the neuron high state) is preceded by an increase in the number of Class E1 and I1 neurons in the neuron high state.

### Topologically-induced inbalance increases sigmoidally for 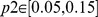


In order to determine the influence of the rewiring parameters in the rewired ring lattice, we varied 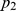 with 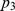 fixed, and *vice-versa*. Figure 7 shows the time-averaged fraction of neurons in Classes E1, I1, E2 and I2 in the neuron high and low states, from 10 realisations of each network for increasing rewiring probabilities. The data shows that increasing 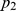 with 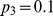 leads to a sigmoidal increase in the fraction of Class 2 neurons that are in the high state. As the fraction of Class 2 neurons saturates for 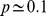, the number of Class 1 neurons in the low state also increases significantly above the benchmark case of no rewiring.

Figure 7 also shows that increasing 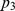 leads to a decrease in the overall number of Class 2 neurons in the neuron high state.

### Topologically-induced bistability in Class 2 induces imbalanced input spike-rates in Class 3

It is also noticeable in Figs. 5 and 6 that some Class E3 neurons also fire at high rates over the same time periods as the localised cluster of highly active Class E2 and I2 neurons. Given that these do not occur in clusters, we hypothesised and confirmed that these cells by chance received a relatively low proportion of their incoming inhibition from the cluster of high firing Class I2 cells. Given that we restrict our attention to network upstates, here we do not classify neurons as being in high, low or neutral states as done above. Instead, based on average spike rates in the random network, a neuron was defined as ‘*highly active*’ if it had more than 4 spikes per second for E neurons and more than 30 spikes per second for I neurons, within an upstate. Note that the maximum possible firing rate is determined by the refractory period, i.e. the maximum rate is 500 spikes per second for E neurons and 1000 spikes per second for I neurons.

**Figure 9:**
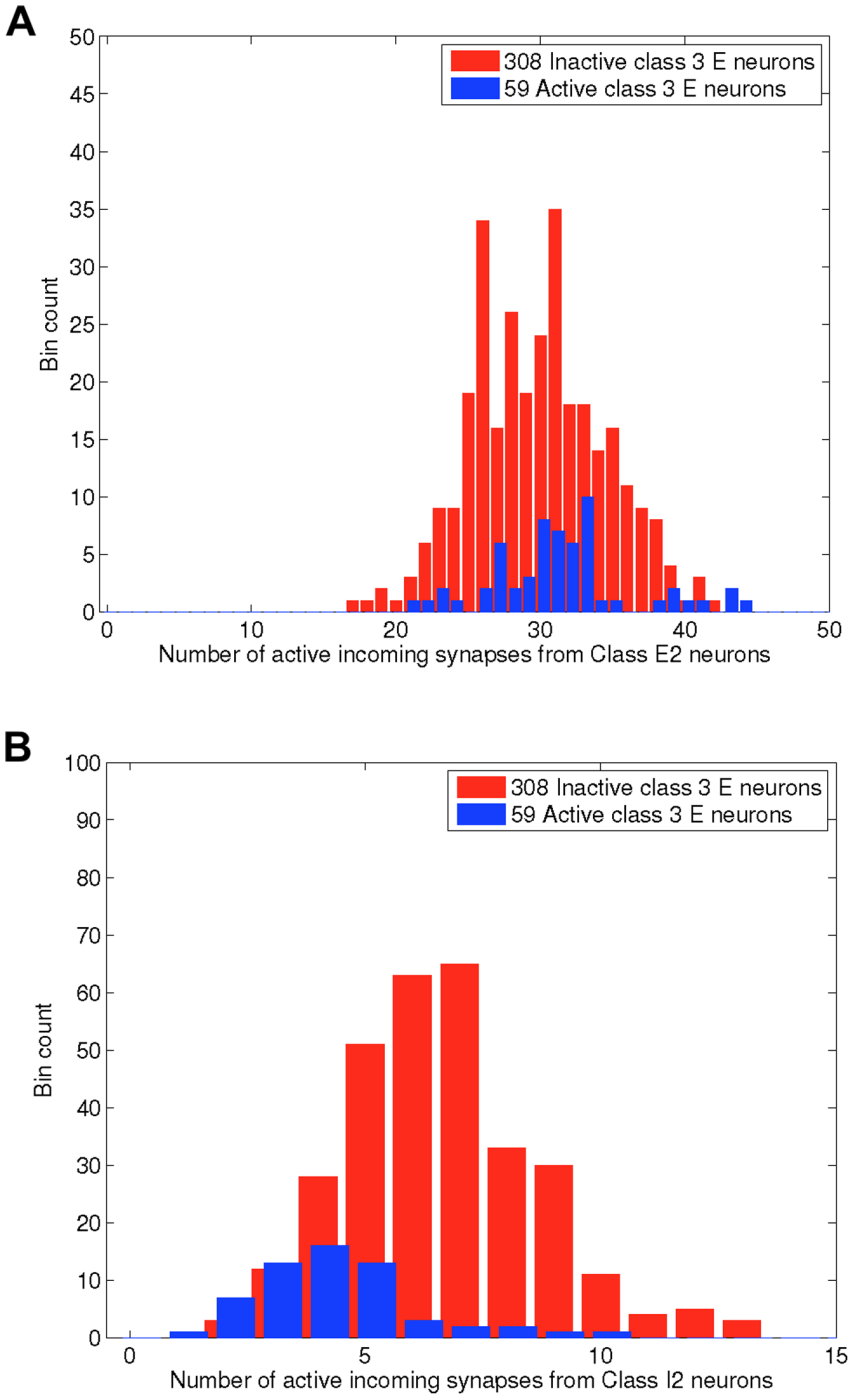
Network upstates create a temporary functional network in which some Class 3 neurons receive an imbalance of excitation and inhibition. This figure shows the ‘functional in-degree’ from different neuron classes to active neurons, from simulations of the rewired ring lattice (with 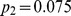 and 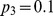). The shown data are from the highly active period between 0.5 and 1.5 seconds shown in Figures 3 and 6. Each subfigure shows the frequency with which both active and inactive Class E3 excitatory neurons (i.e. those that had all incoming synapses rewired), have different numbers of incoming synapses from active Class E2 (subfigure A) and Class I2 neurons (subfigure B; those neurons that had incoming inhibition only rewired). In contrast, in the network downstate, the entire network is included in a functional network, and all neurons have identical numbers of excitatory incoming connections (800) and inhibitory incoming connections (200).

Figure 9 shows histograms of the number of incoming synapses to highly-active and non-highly-active Class E3 neurons, from highly active Class E2 and I2 neurons. The histograms were created from the data shown in Figure 3 between 0.5 and 1.5 seconds.

In Figure 9 we introduced the label *functional in-degree* to describe in-degrees from neurons that are functionally highly active.

The data in Figure 9 indicate that the number of incoming synapses from highly active Class E2 neurons has little correlation with whether Class E3 neurons are highly active. In contrast, the number of incoming synapses from Class I2 neurons are rarely greater than 5 for highly active Class E3 neurons, but likely to be larger than 5 for non-highly-active Class E3 neurons. This suggests that when Class E3 neurons become active during the time that clusters of Class E2 and I2 neurons are active, this is caused by the Class E3 neurons receiving many excitatory spikes from highly active Class E2 neurons, but insufficient numbers of inhibitory spikes from highly active Class I2 neurons, instead receiving most inhibitory synapses from the less active Classes I1 and E3.

### Identifying our structurally-defined neuron classes based on network science metrics

It is of interest to consider how the structurally-defined neuron classes we constructed in our rewired ring lattice network might be identified based on connectivity data alone, i.e. based on statistical features of the adjacency matrix, 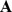. Similar methods will become applicable to real neuronal connectivity data when sufficient connectome data are acquired. Obviously many other ways of classifying neurons according to structure are possible, such as whether or not they participate in modules.

**Figure 10:**
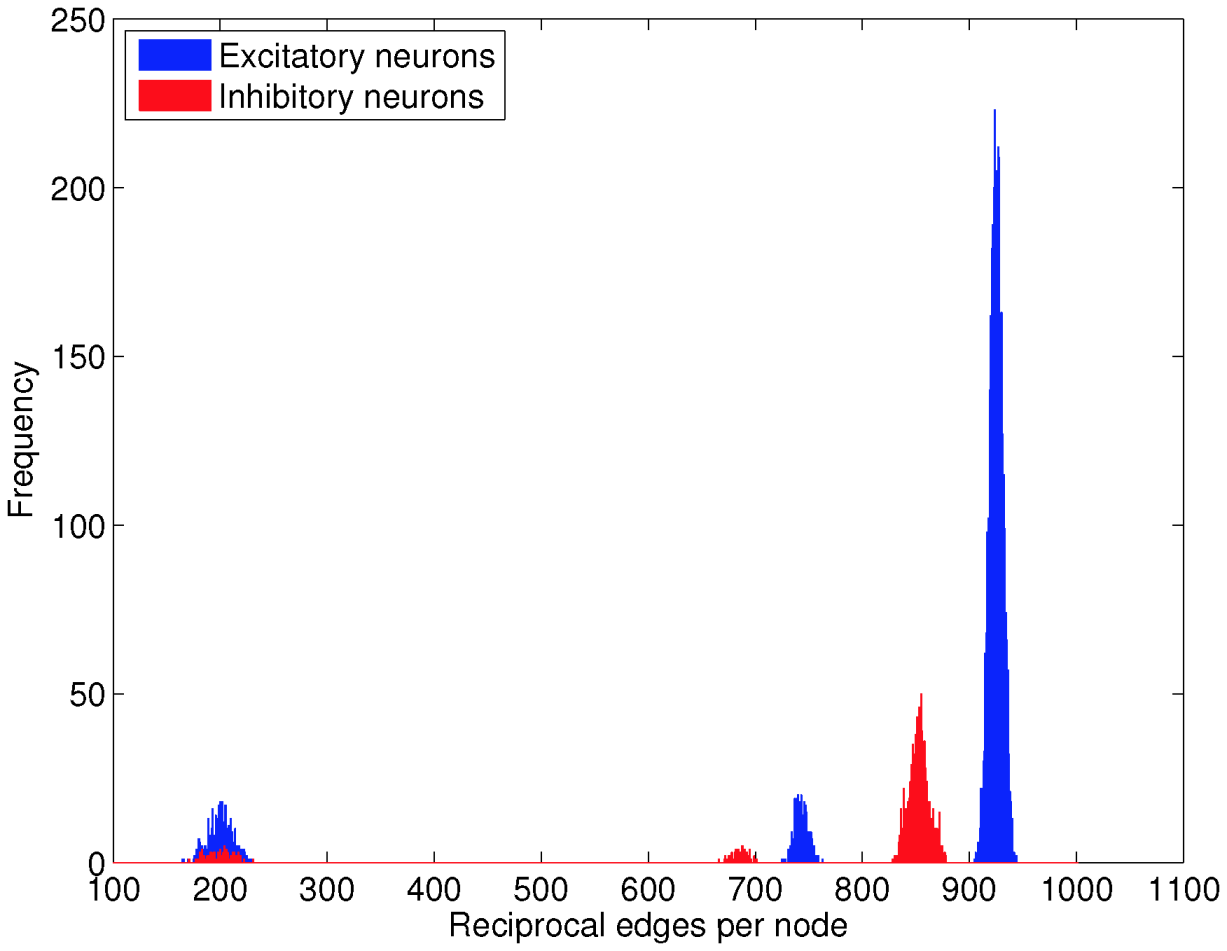
Structurally-defined neuron classes can be identified mathematically from the adjacency matrix. This figure shows histograms of the number of reciprocal connections in which each neuron participates, for the 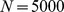 rewired ring lattice (with 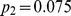 and 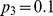) used to obtain the data shown in Figures 1, 3 and 6. There is an obvious classification of these data into three kinds of structurally-defined neuron classes for both excitatory and inhibitory neurons. Indeed, the classification corresponds exactly to the Class 1, Class 2 and Class 3 neurons created during the rewiring procedure.

As was expected based on knowledge of construction of the network, one simple method for identifying structurally-defined classes is to combine information about whether a neuron is excitatory or inhibitory with a count of the number of reciprocally connected neuron pairs in which each neuron participates. Figure 10 shows histograms of such data, and a clear separation between three groups of excitatory neurons and three groups of inhibitory neurons can be seen. Upon checking against information from the network construction, each separate group aligns perfectly with which neurons were chosen for which kind of rewiring.

### Predicting loss of balance based on network science metrics

In the previous section we demonstrated that specific structurally-defined neuron classes in our rewired ring lattice network could be readily identified solely from an adjacency matrix, 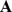. It is, however, a much more difficult proposition to predict, based on the adjacency matrix, (i) loss of globally-balanced excitation and inhibition, due to bistable population rates; and (ii) that different structurally-defined neuron classes that might be identified should exhibit different bistable spiking modes. Nonetheless, in this section we aim to provide some insight into what the topological features of the rewired ring lattice might contribute to this problem.

Above, we also provided evidence that Class E2 and I2 neurons provide the largest contribution to high firing rates during upstates. In the construction of the rewired ring lattice, however, all excitatory neurons, regardless of whether or not they were chosen for rewiring, receive and send on average 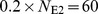 excitatory inputs from/to Class E2. Conversely all excitatory neurons receive and send on average 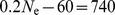 excitatory inputs from/to either Class E1 or Class E3 neurons. Consequently, the number of synaptic connections from one class to another class cannot be the cause of loss of balance and introduction of bistability. We therefore sought to find some other statistical metric that may be a predictor of loss of balance.

One possible avenue to this end is to count the number of positive and negative feedback connections in which each neuron participates. By feedback we mean directed paths on a graph that start and end on the same node. Positive feedback paths are those with an even number of inhibitory edges (including none) and negative feedback paths are those with an odd number of inhibitory edges [66].

#### Positive and negative feedback reciprocal connections do not alone predict loss of balance

**Figure 11:**
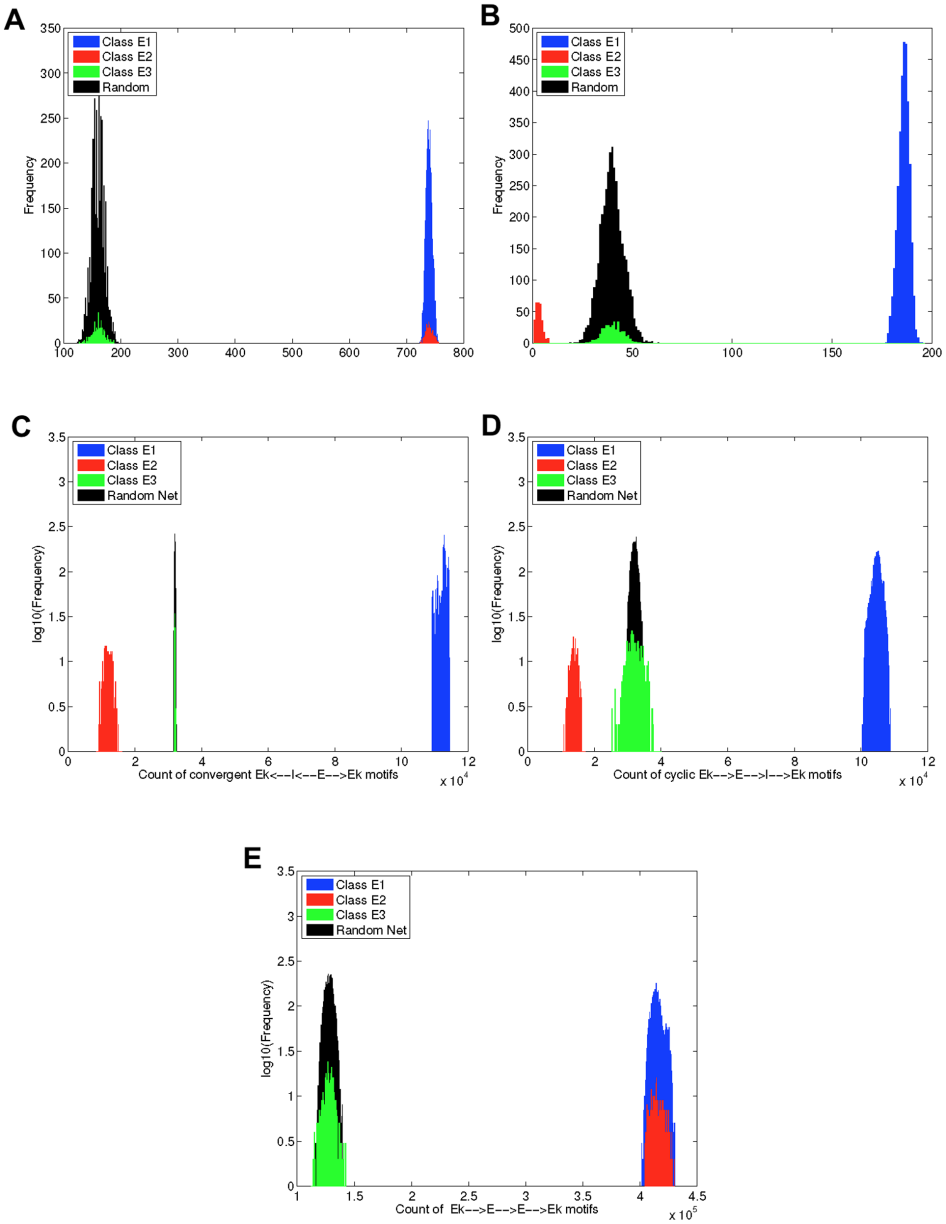
Different structurally-defined neurons participate in different numbers of two and three edge motifs. This figure shows (for the rewired ring lattice with 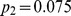 and 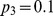) histograms of the number of positive (A) and negative (B) feedback reciprocal connections for excitatory neurons; two types of negative feedback node-referenced three-synapse motif count histograms for excitatory neurons (C) and (D); and the all-excitation positive feedback motif count distribution for excitatory neurons (E). This data illustrates that although Class E2 neurons participate in as many positive feedback connections as E1 neurons, Class E2 excitatory neurons also participate in significantly fewer inhibition-inducing connections than Classes E1 and E3, and than E neurons in the random network. The notation 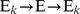 indicates that the motif is one where a reference neuron (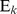) sends synaptic input to an excitatory neuron, which also sends synaptic input to the reference neuron. Notation of the form 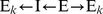 indicates that the motif is one where a reference neuron receives input from a synapse with an inhibitory neuron, which receives input from a synapse with an excitatory neuron, which also sends synaptic input to the reference neuron.

The simplest kind of feedback path is that involving a reciprocally connected pair of nodes (see Figure 10). We therefore examined whether Class E2 neurons exhibit differences in the number of positive and negative reciprocal paths in which they participated. As shown in Figure 11, Class E2 neurons participate in approximately as many positive feedback reciprocal connections as Class E1 neurons. They also participate in many fewer negative feedback reciprocal paths, however, and indeed, in fewer such paths than do E neurons in a random network, or than do Class E3 neurons.

These data suggest that the ratio of positive to negative reciprocal connections may be a predictor of loss of globally balanced excitation and inhibition. When we tested this hypothesis, however, by creating a network that was random other than for artificially introducing higher than random ratios of positive to negative reciprocal connections for some E neurons, we did not observe that those cells had significantly increased firing rates (data not shown). This was the case even when the ratio was made as large as possible within the constraints imposed by not changing in-degrees. Consequently, reciprocal connectivity on its own cannot be a predictor of loss of balance in our model, and it is necessary to consider motifs consisting of at least three nodes or three edges.

#### Three-edge inhibition-inducing motifs occur less frequently for Class E2 nodes

There are three types of motifs that consist of two edges and three nodes in which a particular node can participate (those that converge on the node, those that diverge from the node, and those in which the node is a relay) [21]. When differences between E and I nodes are considered, if the reference node is excitatory, then there are three kinds of convergent and three kinds of divergent motifs, and there are four kinds of relay motifs. When E and I reference nodes are considered, there are 20 such motifs in total [67]. There are many more kinds of motifs that consist of three nodes and three edges in addition to both E and I nodes. It is difficult to identify which, if any, of all these motifs are the best predictor that Class E2 neurons might be a cause of loss of balance. In order to demonstrate that Class E2 neurons do differ statistically from other E neurons in motifs that could play a role, however, we have examined data for two kinds of motifs consisting of three edges and three nodes, where the reference node is an E node.

The aim was to to identify neurons that are more likely to become hyperexcitable, due to participating in fewer inhibition-inducing motifs than other neurons. Both of the mentioned motifs are likely to lead to the reference node becoming less excited. The first is the case where the reference node participates as the convergent node from an E node and an I node, where the E node also connects to the I node (we label this motif as 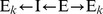, where 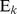 is the reference node). In this case, when the E node fires it excites the reference node, but can then also indirectly inhibit it, if the I node also fires as a consequence. This motif has been studied specifically in [62],[68] (where it referred to as a feed-forward inhibition (FFI) motif) in terms of correlations between activity in the I node and the reference node. The second is a negative feedback cycle where the reference node connects to an E node, which connects to an I node, which connects back to the reference node (we label this motif as 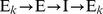, where 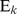 is the reference node). When the reference node fires, it excites the E node, which in turn may excite the I node if it fires. Then if the I node fires, it inhibits the reference node.

Data for both of these motifs are shown in Figure 11. These data show that Class E2 neurons participate in many fewer inhibition-inducing motifs of both kinds than do either Class E1 and E3 neurons, or than do E neurons in a random network. Crucially, both E1 and E2 neurons participate in about the same number of positive feedback cycles involving three nodes, a much larger number than for E neurons in the random network (Figure 11(E)).

## Discussion

### Complex networks can sustain both balanced and unbalanced cortical activity

We have shown that simulations of a population of cortical neurons connected by a complex network topology can exhibit collective dynamics that have not been observed for a simpler version of the same model nor for a random network. Specifically, a randomly rewired ring lattice network with 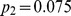 and 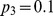 exhibits unbalanced dynamics (Figs. 1 and 3) and also robustly, but stochastically, switches between a high firing state, and a low firing state (Figs. 4 and 7). The average dwell time in each state is of the order of 0.5 seconds, and thus the average frequency of the bistable dynamics is about one hertz. This is despite the complex network maintaining some important properties of a random network, namely recurrent connections and overall unchanged mean in and out degrees.

We note, based on Figure 7, that choosing 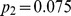 ensures the rewired network is in a region where bistability is most pronounced—small 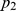 leads to fewer upstates, while larger 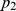 can lead to the network transitioning to the upstate and never leaving it. There is, however, a small range of 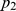 (approximately 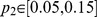) over which transitions in both directions occur robustly for sufficiently long simulation durations. We also note that the effect of increasing 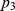 from zero decreases the total number of Class 2 neurons in the neuron high state, and that our data for the selected value of 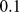 is very similar to the case of 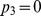.

We further demonstrated (see Figure 9 on differences in ‘functional in-degree counts’) that it may also be necessary to consider the influence of transient functional network topology induced by bistable population dynamics, such as cortical up and downstates (see below), on firing patterns in neurons that are not part of the functional network (i.e. those Class 1 and 3 neurons not participating in network upstates).

Actual *in-vivo* recordings reveal that excitation and inhibition is often (but not always) balanced, and firing rates in individual neurons are usually low and irregular [41]. These properties have been observed in numerous simulations based on random networks. When we previously briefly reported (as part of a broader study that focussed on gamma frequency oscillations) simulation results using similar networks [23], however, we studied a variety of complex network variations not described in the current paper and many of those did not lead to results very different from the deterministic ring lattice or random network, i.e. balance was maintained in most of the variations. We did report, however, that for a single variation similar to that used here, some neurons in the network would begin to fire at much higher average spike rates than those in the random network. The initial aim of the work reported in the current paper was to understand what specific kinds of random rewiring did cause such loss of balanced dynamics.

Our results in the current paper and in [23] together provide evidence that simulations are unlikely to be capable of accurately predicting network structure in real neurobiology. They also suggest, however, that any deviations from the balanced state *in-vivo* could plausibly be due, at least partially, to complex network connectivity features such as the combination of high clustering with inhomogenous motif participation, as in the specific structurally-defined classes we used. Until connectomics methods mature sufficiently to map individual neuron connectivity in large enough cortical regions it will not be possible to test this proposition.

### The mechanism of robust unbalanced bistable dynamics: non-local inhibition received by a local cluster of active excitatory neurons

As shown in Results (see Figures 5, 6, 7 and 8), subnetworks of Class E2 excitatory neurons in our model (i.e. neurons whose incoming inhibition is rewired at the originating end, but their excitatory input is not rewired) fire at high rates during the network upstate. Our explanation for why Class 2 neurons can become highly active is as follows (note that we ignore Class 3 neurons in this discussion, as removing them entirely from the network has negligible impact on bistability).

Each Class 2 neuron receives all its recurrent excitatory inputs from a local subnetwork of highly connected excitatory neurons. In contrast, all its inhibitory inputs are from randomly chosen inhibitory neurons from outside its local vicinity, and the inhibitory neurons those inputs come from do not form such a highly connected subnetwork. In the deterministic ring lattice, and in the downstate of the rewired lattice, if by chance (due to the fluctuation driven nature of the model), a larger than average number of excitatory neurons in a local part of the ring fire within a short space of time, this can lead to transient increased firing in both local excitatory and inhibitory neurons, that quickly dampens down in Class 1 excitatory neurons due to feedback inhibition.

Such an event, however, has a different impact on Class 2 neurons, giving rise to the upstate in the following sequence, which is consistent with data shown in Figures 6 and 7:

- Step 1: If enough Class E2 neurons in the local vicinity of a fluctuation-driven rise in excitation are recruited to spike (perhaps due to spatially correlated firing, as shown for the unrewired ring lattice in Figure 2), then positive feedback mechanisms keep them firing, since these neurons do not receive any local inhibition.
- Step 2: The onset of the neuron high state in these Class E2 neurons triggers the neuron high state in local I2 neurons, which are also not inhibited due to receiving no local inhibition.
- Step 3: These high firing local I2 neurons quench all spiking activity in local E1 and I1 neurons, putting them into the neuron low state, and also in non-local E2 and I2 neurons, and therefore the only avenue for inhibition to come back to the high state neurons is from nonlocal I1 neurons, and the rate of this inhibition is typically too slow to dampen spiking.

Note that non-local E1 and I1 neurons are hardly affected by the transition to the network upstate, and continue to fire at rates comparable to the lattice network, and the downstate in the rewired network. Note also that we verified the assertions in Steps 2 and 3 by analysing the mean conductances of all classes of neuron. During an upstate, the mentioned local E1 and I1 neurons, and non-local E2 and I2 neurons all enter a state where they have a much higher than average inhibitory conductance, so that they are very unlikely to fire. This entry to the neuron low state can be seen in Figure 6, where there are few, if any, spikes for E1 and I1 neurons in the location of network upstates, and similarly in E2 and I2 neurons outside the location of upstates. It is also consistent with Figure 7, which shows an increase in the number of neurons in the neuron low state as the number of high state neurons increases.

We now consider the transitions from the network upstate to the network downstate. Our simulation data (Figure 8) suggest that most such transitions are preceded by a transient (driven by fluctuations in external input) increase in excitatory spiking by Class E1 neurons that are not local to the highly active Class 2 neurons. This indicates the following mechanism:

- Step 1: During an upstate, some E1 and I1 neurons that are nonlocal to the high state neurons begin to fire transiently.
- Step 2: The firing of these non-local I1 neurons can send higher than average non-local inhibition to both the high state E2 and I2 neurons. The transient firing in non-local E1 neurons does not significantly affect the E2 and I2 neurons, since it was only the incoming inhibitory connections that were rewired in Class 2 neurons to originate from non-local regions.
- Step 3: If this additional inhibition is sufficiently large, it reduces the membrane potential of high state Class E2 excitatory neurons, thus shutting down the network upstate.

Following the transition from a network upstate to a downstate, the network is in the same state as the lattice network. Hence, the same conditions exist that gave rise to the first upstate.

Overall, in our model it is the interaction between clustering, and non-local inhibition that gives rise to bistable population dynamics. As shown in Figure 2, clustering alone is insufficient to give rise to bistability. The fact that the rewired network was initially a ring lattice results in the possibility that the upstate can be triggered at many different locations around the ring, albeit biased by where Class 2 nodes are most densely located. This suggests a general principle that might be replicated in modular or other kinds of spatially clustered networks involving both long and short range connections.

Our findings on bistable population activity arising from topological network changes are superficially similar to those reported in [27], where similar bistable dynamics were observed in a network akin to the fourth network topology (embedded modular) that we studied. We did not, however, observe robust bistable dynamics for the embedded modular network. As shown in a typical example in Figure S2, after some transient initial bistability, activity in the modular network switches to a sustained highly active state, and does not switch back to the low firing state.

A possible explanation for the lack of robust sustained bistability in the embedded modular network in our study may be that we used several different modelling assumptions in comparison with the models of [27]. First, the neurons in their model were activated by a constant supratheshold voltage ‘bias’, which is equivalent to a constant injected current. These were chosen randomly for each neuron, resulting in a heterogenous population of neurons, where there is a range of firing rates in the absence of recurrent feedback. In contrast, individual neurons of each type in our model are identical, and each has an identical firing rate in response to noisy external drive in the absence of recurrent feedback. Second, their model considers a modular network only for excitatory neurons, with a clustering coefficient close to that of a random network, and all other connectivity is random, whereas ours considers very high clustering of both excitatory and inhibitory neurons. Third, and most crucially, we introduce structurally-defined neuron classes into our network model such that different classes co-exist in a local cluster, and these do not exist in [27].

A likely reason why we did not observe bistability in the embedded modular network, and only loss of balanced excitation and inhibition, is that after the transition to the upstate within a module, all excitatory cells in the module participate in the upstate. Although this excitation is spread to all inhibitory neurons, the extent of excitation in a module is too great for inhibition to damp down. It is possible that if we biased our excitatory neurons by including a random spread of membrane potential threshold values, in order to make our neurons more like those of [27], we would see see bistability induced only in neurons with lower thresholds.

When comparing the results of [27] to those of our rewired lattice network, the second and third differences between the models give rise to bistability with different characteristics, since we found that only our Class 2 neurons become highly active; these neurons do not differ in their intrinsic dynamics, but only in the distribution of the structural motifs in which they participate.

### Can complex network metrics predict transitions between upstates and downstates?

We found that reciprocal connectivity alone cannot predict loss of balance in the rewired ring lattice, because artificially altered reciprocal connectivity in an otherwise random network does not lead to loss-of-balance/bistability. In the context of the mechanistic explanation above, this is not surprising. We have also shown for this network that Class E2 and I2 neurons may fire at high rates during upstates (see Figs. 5 and 6), and that Class E2 neurons participate in some kinds of inhibition-inducing three-edge motifs many fewer times than do neurons in a random network, or than do Class E1 or E3 neurons (Figure 11). We suggest, however, based on the reciprocal connectivity result, and the bistability mechanism, that it is unlikely that contriving a random network with artificially altered three-edge motif counts for some excitatory neurons will result in loss of balance.

It is, however, likely that the rates of transitions from upstates to/from downstates (which determine the mean dwell time of each state) is related to the motif participation counts for Class E2 and I2 neurons. A potentially feasible approach is to study the correlations in spiking activity induced by our different classes just prior to a transition. Indeed, our mechanistic explanation above suggests studying the influence of uncorrelated negative feedback in E2 neurons in a manner similar to work in [69]. It may well be the case that mathematical theory regarding the relationship between structural motifs and spike correlations introduced by [19],[62],[67],[68],[70] can be generalised to the complex network case. Additionally, bistable dynamics have been studied mathematically in balanced networks similar to that studied here but without complex network topology [71]. It is possible that this theory could be adapted to networks with high, but inhomogeneous, clustering, like that in our model.

We have also briefly shown that what we call functional in-degree is a predictor of unbalanced activity in Class 3 neurons (Figure 9). This suggests that studying functional networks that exist only transiently may be a useful tool in predicting the influence of topology on dynamics.

Any suitable predictor of loss of globally balanced excitation and inhibition and bistable population activity should enable construction of a network in which features that produce loss of balance exist, but which is otherwise random. Finding a suitable metric, and network construction algorithm is left for future work, but we note that an approach of this kind was studied by [68] in terms of how increased FFI motifs embedded in a random network tended to have a stabilising influence. Here we found that excitatory neurons that participate in fewer FFI motifs within a complex network are most active.

The subnetwork of excitatory neurons and their recurrent synapses created by [27] is, by construction, a prototypical modular network like that of [72], since every excitatory neuron belongs to a ‘community.’ Each neuron within a distinct module or community connects with greater probability to neurons within that community than to those outside it. In this network there is only one class of structurally-defined excitatory neuron, and one class of structurally-defined inhibitory neuron, in the sense that we use this term in this paper. It is, of course, possible to classify all excitatory neurons in each module as belonging to a structurally-defined class, but this classification can then not be carried out by analysing individual neurons and their incoming and outgoing links in isolation from the rest of the network.

We also point out that we do not have structural modularity in our network. The standard network science definition of modularity [73], is such that groups of neurons are assigned to a module if the connection density between the neurons in that module is significantly greater than the connection density amongst edges into or out of the module. As mentioned above, in the rewired ring lattice, the mean degree into a node of any class, from a given class, is always 20% of the size of the given class. A similar constraint holds for out-degree.

### Could clustering and inhomogeneous motif participation cause empirically-observed cortical ‘up and down’ states or hyperexcitability?

It has been proposed previously that connectivity is likely to be a useful means of classifying neurons when it comes to understanding their role in information processing [2]. Given that we have found that neuron models that are identical in terms of their membrane and synaptic dynamics can be grouped into classes entirely based on structural connectivity differences (and these different classes exhibit differences in their firing characteristics) provides strong support to this proposition.

It has further been suggested [2] that disorders in connectivity topology (‘connectopathies’) may lead to brain disorders, such as epilepsy. The results we report here lead us to speculate that transient or bistable loss of E-I balance in neuronal dynamics can lead to hyper-excitation that manifests itself as epileptic seizures.

Even though the rewired ring lattice is unlikely to exactly resemble real neocortical structure, there is strong experimental evidence for clustering among excitatory neurons, as discussed by [27], and predictions of clustering based on spatial considerations have arisen from theoretical work [74].

It has also been reported that small groups of highly active neurons can dominate firing within neocortical networks [75]. Our simulation results are consistent with these experimental findings, in the sense that our Class 2 neurons are those that participate most in the upstates, but form only a small percentage (7.5%) of the network, and thus they form a small subnetwork of highly active neurons. It is possible that the results of [75] could also be explained in terms of structurally-defined neuron classes akin to those in our model, were the necessary connectivity data available.

Our results are also reminiscent of so called cortical up and downstates [76], and it is possible that more complete understanding of their origin and function will require studies of whether they may arise from the dynamics of specific structurally-defined neuron classes and complex network connectivity that depends on node and edge classes. Various theories about both origin and function exist [77], but none, to our knowledge are based on structurally-defined neuron classes.

### Possible origins of heterogenous connectivity structure

Synaptic connectivity between neurons is known to vary over time due to structural plasticity [78]. This could be one mechanism by which complex structure like that discussed in this paper might arise. Another possibility is that it is also known that synaptic efficacies vary greatly. For example experimental findings on the distribution of efficacies has been fitted to a power-law [51], meaning that most efficacies are small and only a small number are large. Given this heterogeneity in connectivity, it is plausible that a model with equivalent dynamics to that studied here could be devised that has random connectivity, but heterogenous efficacy distributions, such that the larger weights are distributed in a complex manner different from the actual connections.

## Supporting Information

Figure S1 **Raster plot of spikes in a single 5.1s simulation for the random network.** Data for excitatory neurons are shown in A and data for inhibitory neurons in B. Shown underneath the raster plots are bar plots of the total number of spikes in each simulation time step (0.05 ms). Shown to the right of the raster plots are bar plots of the total number of spikes in each neuron over the entire 5.1 seconds.
(TIFF)

Figure S2 **Raster plot of spikes in a single 5.1s simulation for the embedded modular network.** Data for excitatory neurons are shown in A and data for inhibitory neurons in B. Shown underneath the raster plots are bar plots of the total number of spikes in each simulation time step (0.05 ms). Shown to the right of the raster plots are bar plots of the total number of spikes in each neuron over the entire 5.1 seconds.
(TIFF)

Figure S3 **LFP for networks realisation 1.** Data for the model LFP at each time step of a simulation, for five independent simulation runs, for the same single realisation of a network. Green traces show data for the rewired lattice and blue traces show data for the random network. Red data shows data for a deterministic ring lattice—this data is the same for each plot. The same 5 realisations of input spikes to each neuron were applied in each network type, and the data for the 5 input spike train realisations are shown in the subfigures.
(TIFF)

Figure S4 **LFP for networks realisation 2.** Data for the model LFP at each time step of a simulation, for five independent simulation runs, for the same single realisation of a network. Green traces show data for the rewired lattice and blue traces show data for the random network. Red data shows data for a deterministic ring lattice—this data is the same for each plot. The same 5 realisations of input spikes to each neuron were applied in each network type, and the data for the 5 input spike train realisations are shown in the subfigures.
(TIFF)

Figure S5 **LFP for networks realisation 3.** Data for the model LFP at each time step of a simulation, for five independent simulation runs, for the same single realisation of a network. Green traces show data for the rewired lattice and blue traces show data for the random network. Red data shows data for a deterministic ring lattice—this data is the same for each plot. The same 5 realisations of input spikes to each neuron were applied in each network type, and the data for the 5 input spike train realisations are shown in the subfigures.
(TIFF)

Figure S6 **LFP for networks Realisation 4.** Data for the model LFP at each time step of a simulation, for five independent simulation runs, for the same single realisation of a network. Green traces show data for the rewired lattice and blue traces show data for the random network. Red data shows data for a deterministic ring lattice—this data is the same for each plot. The same 5 realisations of input spikes to each neuron were applied in each network type, and the data for the 5 input spike train realisations are shown in the subfigures.
(TIFF)

Figure S7 **LFP for networks realisation 5.** Data for the model LFP at each time step of a simulation, for five independent simulation runs, for the same single realisation of a network. Green traces show data for the rewired lattice and blue traces show data for the random network. Red data shows data for a deterministic ring lattice—this data is the same for each plot. The same 5 realisations of input spikes to each neuron were applied in each network type, and the data for the 5 input spike train realisations are shown in the subfigures.
(TIFF)
