# Supplementary figures and images for: Small Modifications to Network Topology Can Induce Stochastic Bistable Spiking Dynamics in a Balanced Cortical Model
Source: PLoS One. 2014 Apr 17;9(4):e88254. doi: 10.1371/journal.pone.0088254 (PMC3990528; doi:10.1371/journal.pone.0088254)

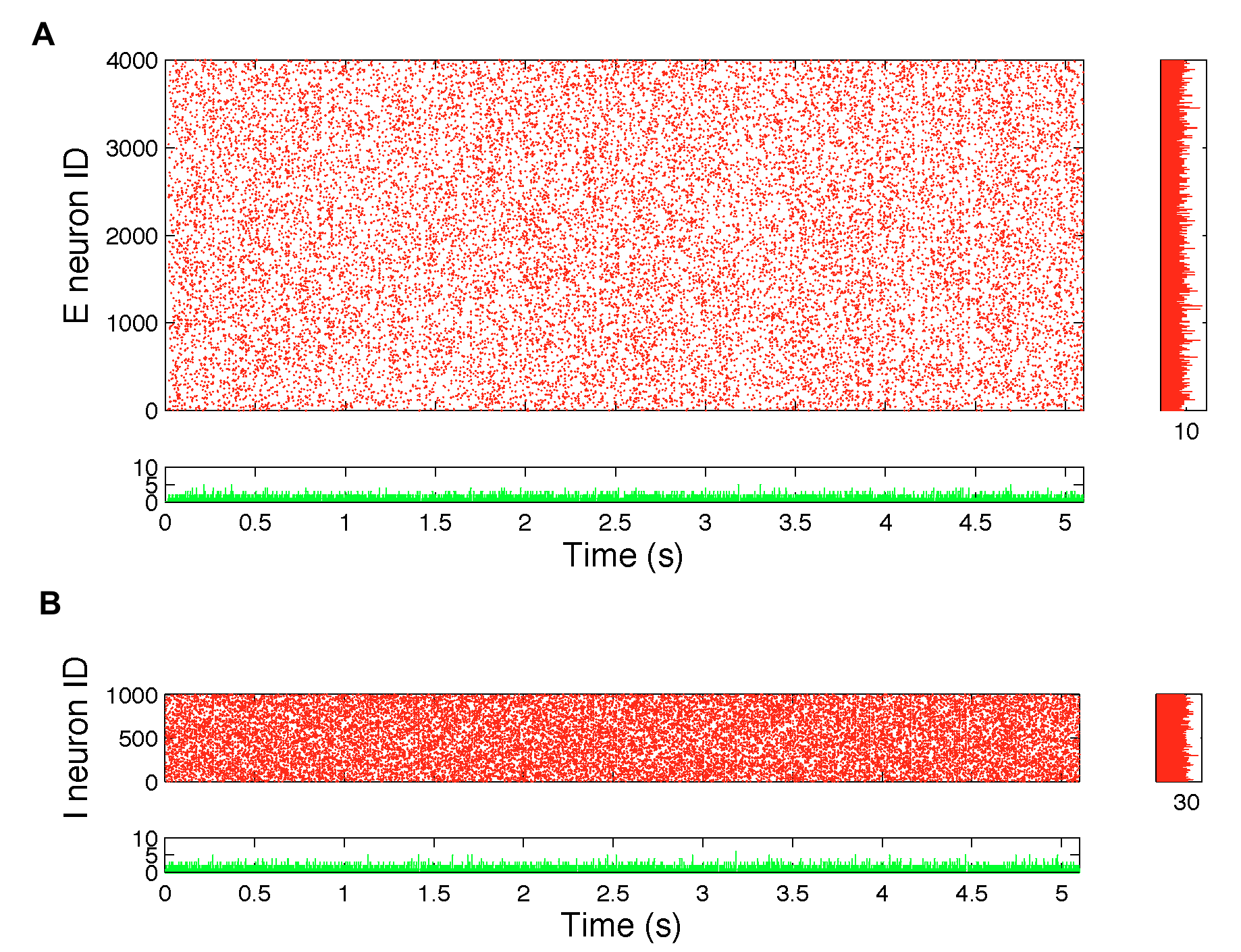

Supplement: Figure S1 — Raster plot of spikes in a single 5.1s simulation for the random network. Data for excitatory neurons are shown in A and data for inhibitory neurons in B. Shown underneath the raster plots are bar plots of the total number of spikes in each simulation time step (0.05 ms). Shown to the right of the raster plots are bar plots of the total number of spikes in each neuron over the entire 5.1 seconds. (TIFF) [file pone.0088254.s001.tif]

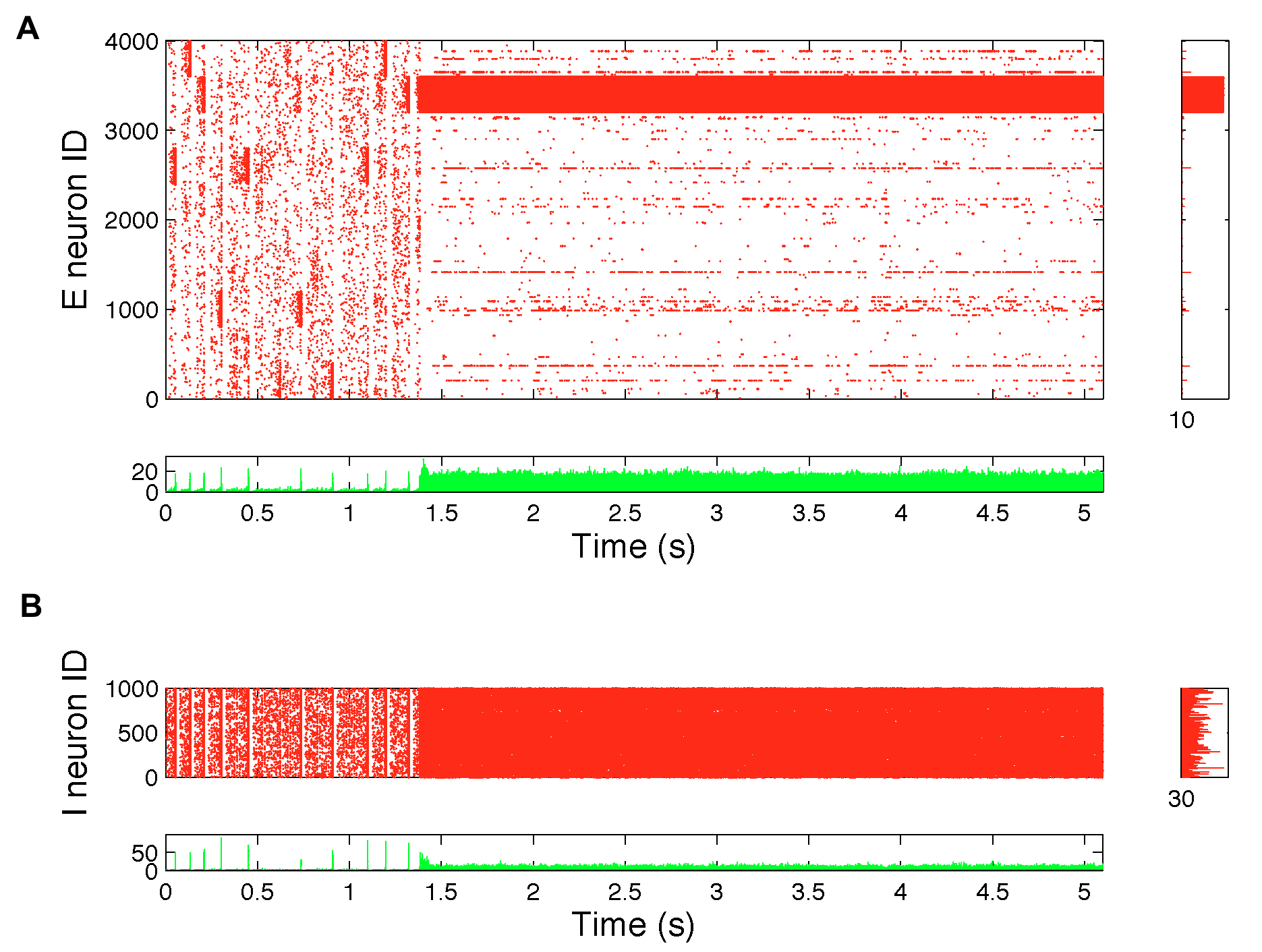

Supplement: Figure S2 — Raster plot of spikes in a single 5.1s simulation for the embedded modular network. Data for excitatory neurons are shown in A and data for inhibitory neurons in B. Shown underneath the raster plots are bar plots of the total number of spikes in each simulation time step (0.05 ms). Shown to the right of the raster plots are bar plots of the total number of spikes in each neuron over the entire 5.1 seconds. (TIFF) [file pone.0088254.s002.tif]

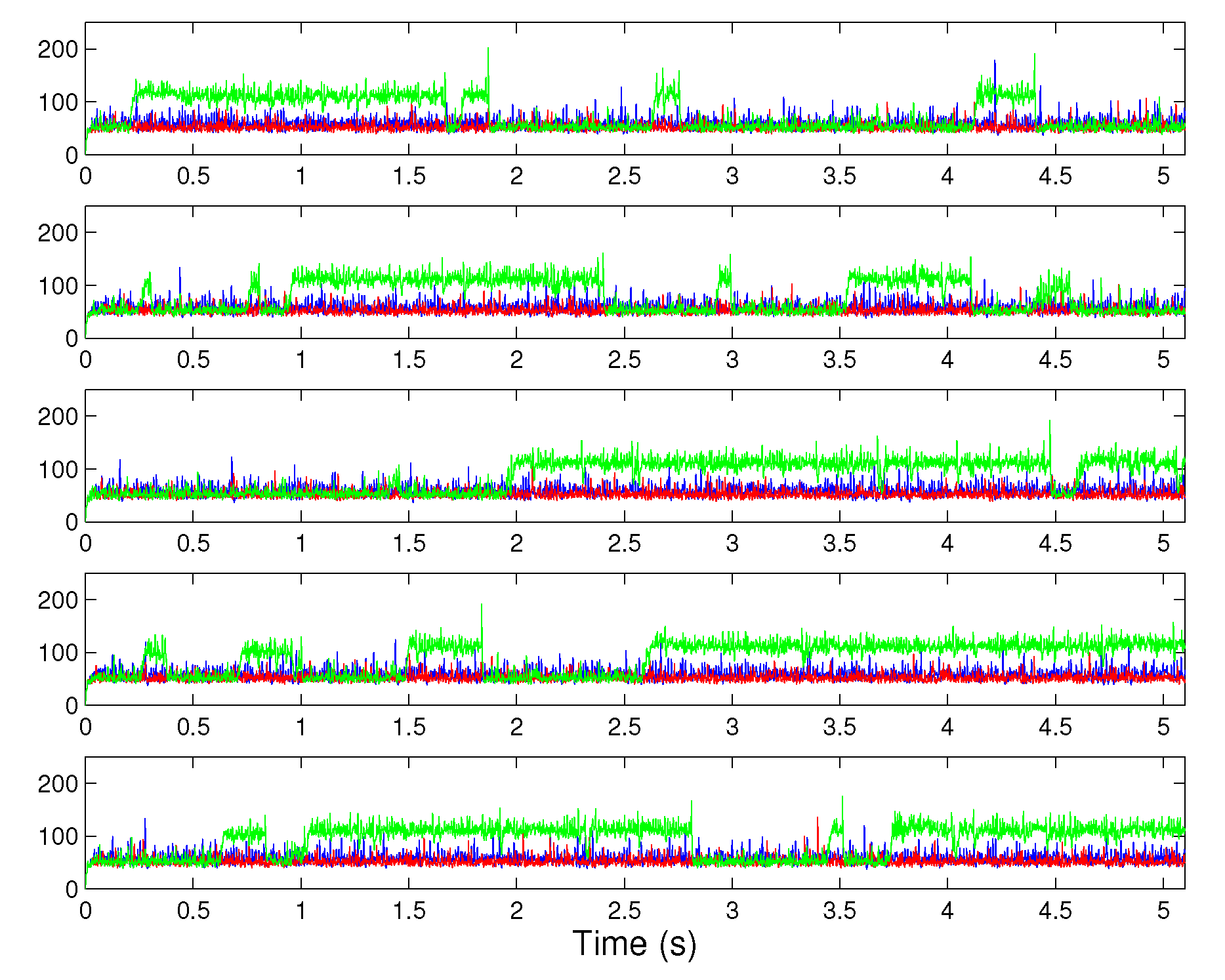

Supplement: Figure S3 — LFP for networks realisation 1. Data for the model LFP at each time step of a simulation, for five independent simulation runs, for the same single realisation of a network. Green traces show data for the rewired lattice and blue traces show data for the random network. Red data shows data for a deterministic ring lattice—this data is the same for each plot. The same 5 realisations of input spikes to each neuron were applied in each network type, and the data for the 5 input spike train realisations are shown in the subfigures. (TIFF) [file pone.0088254.s003.tif]

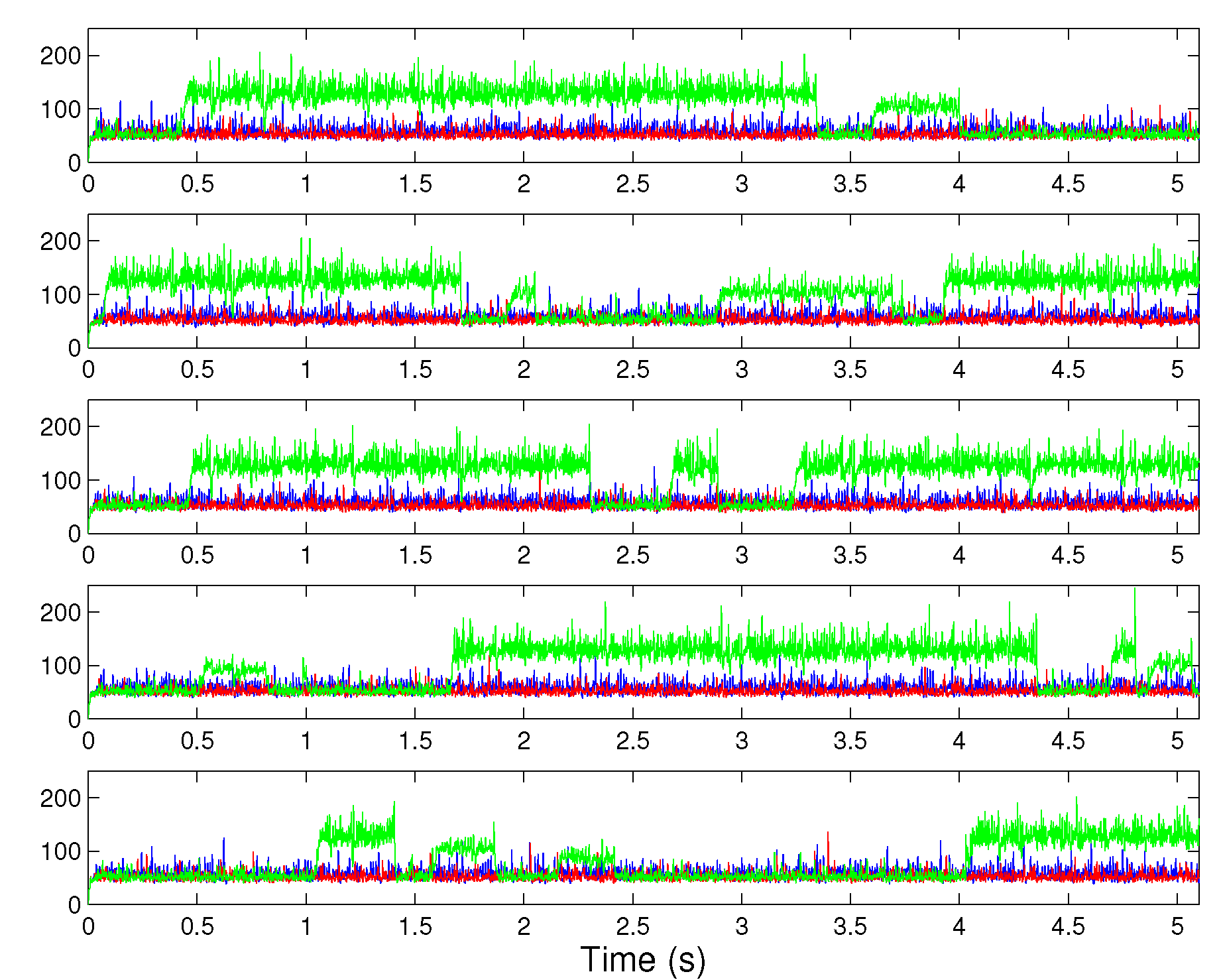

Supplement: Figure S4 — LFP for networks realisation 2. Data for the model LFP at each time step of a simulation, for five independent simulation runs, for the same single realisation of a network. Green traces show data for the rewired lattice and blue traces show data for the random network. Red data shows data for a deterministic ring lattice—this data is the same for each plot. The same 5 realisations of input spikes to each neuron were applied in each network type, and the data for the 5 input spike train realisations are shown in the subfigures. (TIFF) [file pone.0088254.s004.tif]

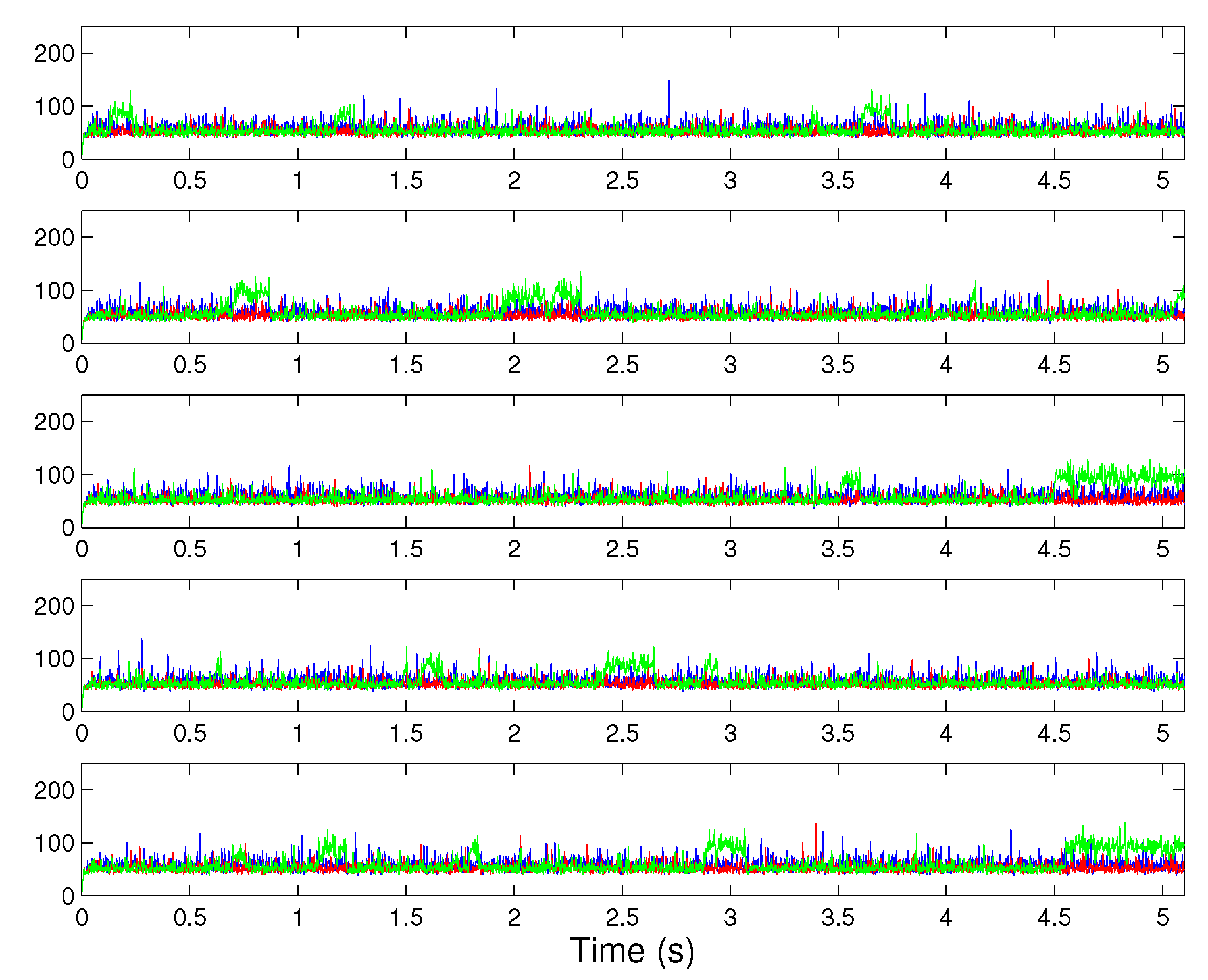

Supplement: Figure S5 — LFP for networks realisation 3. Data for the model LFP at each time step of a simulation, for five independent simulation runs, for the same single realisation of a network. Green traces show data for the rewired lattice and blue traces show data for the random network. Red data shows data for a deterministic ring lattice—this data is the same for each plot. The same 5 realisations of input spikes to each neuron were applied in each network type, and the data for the 5 input spike train realisations are shown in the subfigures. (TIFF) [file pone.0088254.s005.tif]

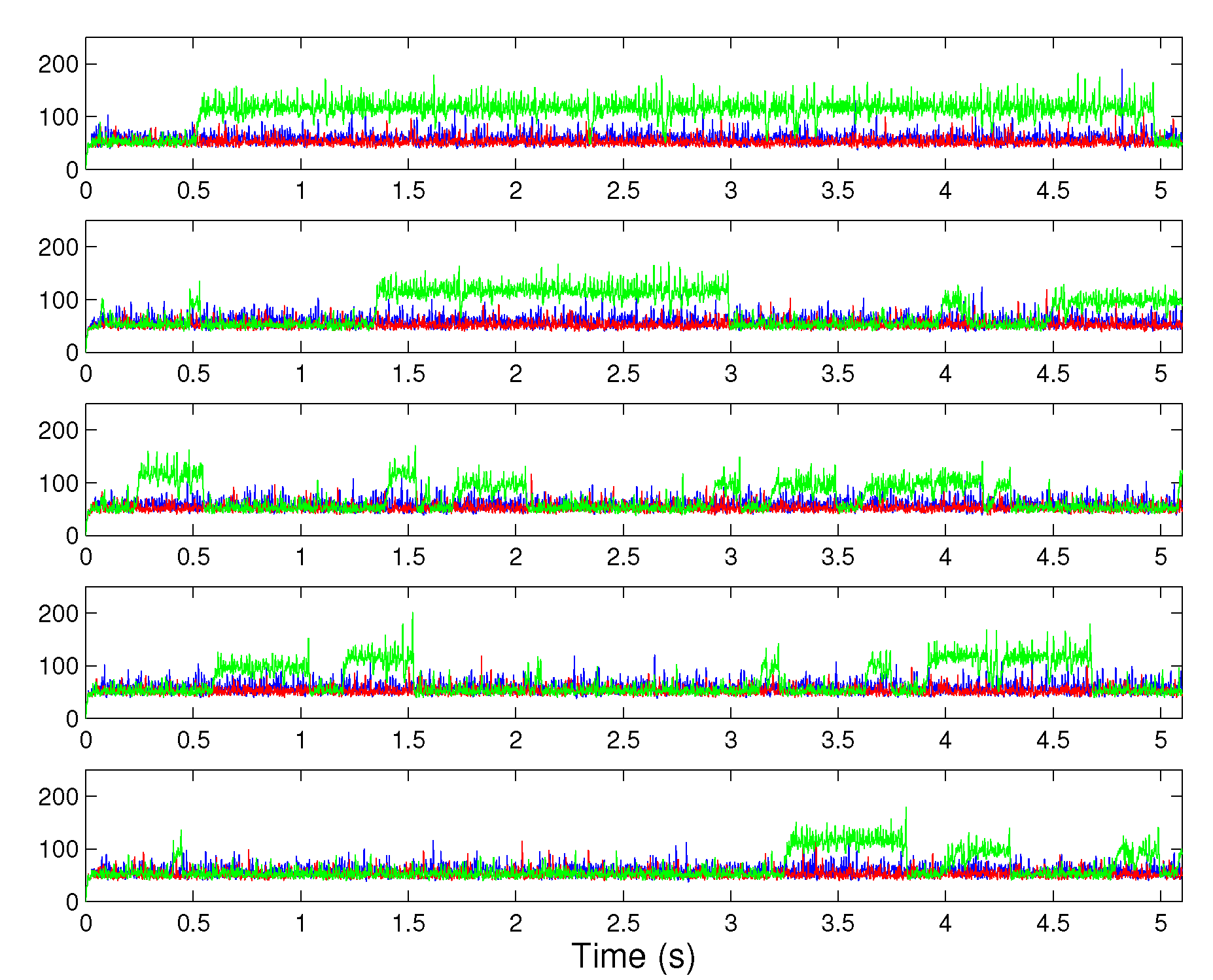

Supplement: Figure S6 — LFP for networks Realisation 4. Data for the model LFP at each time step of a simulation, for five independent simulation runs, for the same single realisation of a network. Green traces show data for the rewired lattice and blue traces show data for the random network. Red data shows data for a deterministic ring lattice—this data is the same for each plot. The same 5 realisations of input spikes to each neuron were applied in each network type, and the data for the 5 input spike train realisations are shown in the subfigures. (TIFF) [file pone.0088254.s006.tif]

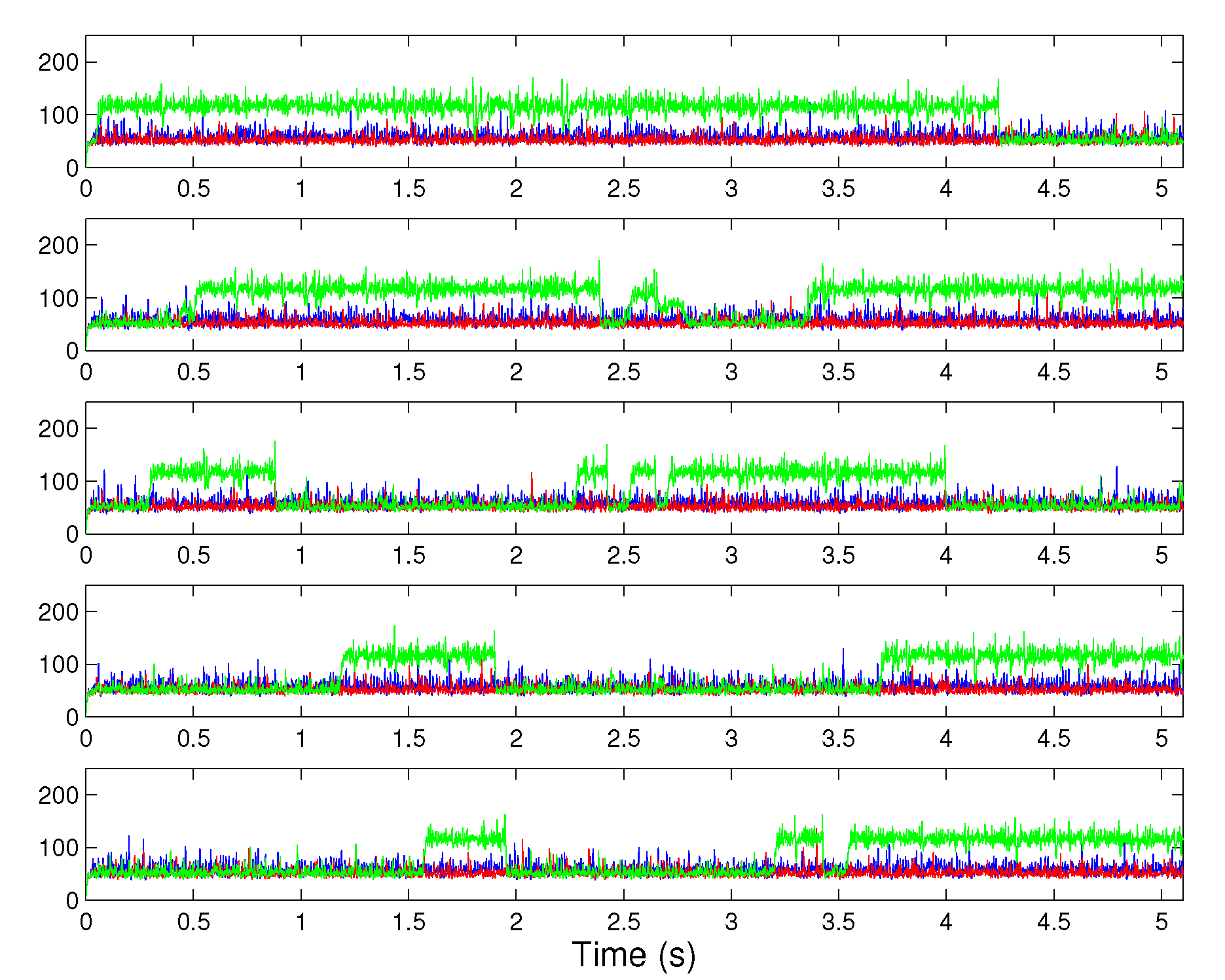

Supplement: Figure S7 — LFP for networks realisation 5. Data for the model LFP at each time step of a simulation, for five independent simulation runs, for the same single realisation of a network. Green traces show data for the rewired lattice and blue traces show data for the random network. Red data shows data for a deterministic ring lattice—this data is the same for each plot. The same 5 realisations of input spikes to each neuron were applied in each network type, and the data for the 5 input spike train realisations are shown in the subfigures. (TIFF) [file pone.0088254.s007.tif]
